# Analysis of small RNAs revealed differential expressions during pollen and embryo sac development in autotetraploid rice

**DOI:** 10.1186/s12864-017-3526-8

**Published:** 2017-02-06

**Authors:** Xiang Li, Muhammad Qasim Shahid, Juan Xia, Zijun Lu, Na Fang, Lan Wang, Jinwen Wu, Zhixiong Chen, Xiangdong Liu

**Affiliations:** State Key Laboratory for Conservation and Utilization of Subtropical Agro-bioresources, South China Agricultural University, Guangzhou, 510642 China

**Keywords:** Female gametophyte, Male gametophyte, MicroRNAs, Polyploid, phasiRNAs, TEs-siRNAs

## Abstract

**Background:**

Partial pollen and embryo sac sterilities are the two main reasons for low fertility in autotetraploid rice. Our previous study revealed that small RNAs changes may associate with pollen fertility in autotetraploid rice. However, knowledge on comparative analysis between the development of pollen and embryo sac by small RNAs in autotetraploid rice is still unknown. In the present study, WE-CLSM (whole-mount eosin B-staining confocal laser scanning microscopy) and high-throughput sequencing technology was employed to examine the cytological variations and to analyze small RNAs changes during pollen and embryo sac development in autotetraploid rice compared with its diploid counterpart.

**Results:**

A total of 321 and 368 differentially expressed miRNAs (DEM) were detected during pollen and embryo sac development in autotetraploid rice, respectively. Gene Ontology enrichment analysis on the targets of DEM associated with embryo sac and pollen development revealed 30 prominent functional gene classes, such as cell differentiation and signal transduction during embryo sac development, while only 7 prominent functional gene classes, such as flower development and transcription factor activity, were detected during pollen development in autotetraploid rice. The expression levels of 39 DEM, which revealed interaction with meiosis-related genes, showed opposite expression patterns during pollen and embryo sac development. Of these DEM, *osa-miR1436_L + 3_1ss5CT* and *osa-miR167h-3p* were associated with the female meiosis, while *osa-miR159a.1* and *osa-MIR159a-p5* were related with the male meiosis. 21 nt-phasiRNAs were detected during both pollen and embryo sac development, while 24 nt-phasiRNAs were found only in pollen development, which displayed down-regulation in autotetraploid compared to diploid rice and their spatial-temporal expression patterns were similar to *osa-miR2275d*. 24 nt TEs-siRNAs were found to be up-regulated in embryo sac but down-regulated in pollen development.

**Conclusion:**

The above results not only provide the small RNAs changes during four landmark stages of pollen and embryo sac development in autotetraploid rice but also have identified specifically expressed miRNAs, especially meiosis-related miRNAs, pollen-specific-24 nt-phasiRNAs and TEs-siRNAs in autotetraploid rice. Together, these findings provide a foundation for understanding the effect of polyploidy on small RNAs expression patterns during pollen and embryo sac development that may lead to different abnormalities in autotetraploid rice.

**Electronic supplementary material:**

The online version of this article (doi:10.1186/s12864-017-3526-8) contains supplementary material, which is available to authorized users.

## Background

Autotetraploid rice is a useful germplasm resource generated by colchicine-mediated chromosome doubling to achieve biological advantages over the diploid counterpart, such as strong hybrid vigor, higher resistance against the abiotic and biotic stresses, and the potential of the biomass production [1, 2]. However, autotetraploid rice has poor seed set, which become the biggest bottleneck and the major barrier in commercial production [3]. Partial pollen and embryo sac sterilities are the two important factors that lead to low seed set in autotetraploid rice [4–6].

Abnormal chromosome behaviors and microsporogenesis were the main cytological reasons for low pollen fertility in autotetraploid rice [6]. Moreover, we also found that abnormal microtubule organization also cause low fertility in autotetraploid rice [7]. Recently, transcriptome analysis revealed that the differential expressions of the meiosis-related or meiosis stage-specific genes may disturb the chromosome behavior during pollen development of autotetraploid rice [6]. In addition to pollen, embryo sac development is also crucial for seed set, and some abnormalities, such as embryo sac degeneration, no egg cells and abnormal position and number of polar nuclei resulted in no embryo or endosperm formation in seeds of autotetraploid rice [4]. These findings indicated that abnormal embryo sac had a strong effect on the fertility of autotetraploid rice. Recently, genome-wide gene expression profiles in ovules development have been reported and they found that dynamic gene expressions may play crucial roles during female gametogenesis in rice [8, 9]. In addition, small RNA sequencing showed that dynamic changes in the expression patterns of miRNA and some specific differentially expressed miRNAs may cause pollen sterility in autotetraploid rice [10].

Small RNAs, the tiny size non-coding RNAs, which are 19-25 nt in length, play major roles in many biological processes, including plant reproduction [11–14]. The small RNAs can be classified into two major types, one is microRNA (miRNA), and the other is small interfering RNA (siRNA). miRNAs, as negative regulators, modulate plant gene expression by cleavage and decay of target transcripts involved in rice pollen development [15–17] and grain filling [18, 19]. Some novel miRNAs have been reported to regulate the pollen and spikelet fertility, such as *miR159*, *miR172*, *miR319* and *miR397* [20–23]. The *miR159*, acts as the posttranscriptional factor, targeted the GAMYB-like genes (*MYB33* and *MYB65*) and restricted the expression of these genes during anther development in *Arabidopsis* [20]. *miR167* is essential for anther and ovule fertility in *Arabidopsis*, and the loss of *miR167* expand the expression of *ARF6* and *ARF8*, and cause abnormalities during ovule and anther development [24]. Furthermore, *miR2118* and *miR2275* are the most important miRNA families involved in the generation of male-specific 21- and 24-nt phasiRNAs (phased small-interfering RNAs), respectively, and some studies suggest that they are essential for male meiosis and pollen development [25, 26]. siRNAs are produced from the transposable elements (TEs) and usually 24-nt in length that guide DNA methylation and histone modifications for transcriptional gene silencing. Transcriptome studies revealed that high expression of TEs have harmful effects on the *Arabidopsis* meiocytes [27]. Additionally, the 24 nt-siRNA abundance related to DNA methylation of class II transposable elements can suppress the expression of nearby genes in autotetraploid rice [28].

Although some information about the expression patterns of small RNAs related with pollen development are available, the expression patterns of small RNAs associated with embryo sac development in autotetraploid rice are still poorly understood. In the present study, we planned to investigate the expression levels of small RNAs during embryo sac development compared to pollen development by using an autotetraploid rice, 02428-4x and its diploid counterpart, 02428, which is a famous diploid rice variety harboring *S*_*5*_^*n*^ gene that can overcome the *indica-japonica* hybrid sterility. 02428-4x was developed from the chromosome doubling of 02428 and was self-crossed for more than 25 generations at our farm. We sought to distinguish the small RNAs changes in autotetraploid and diploid rice, and to determine the relationships between gene expression profiles and spikelet sterility.

## Results

### Cytological observations on pollen and embryo sac development in autotetraploid rice

**Fig. 1:**
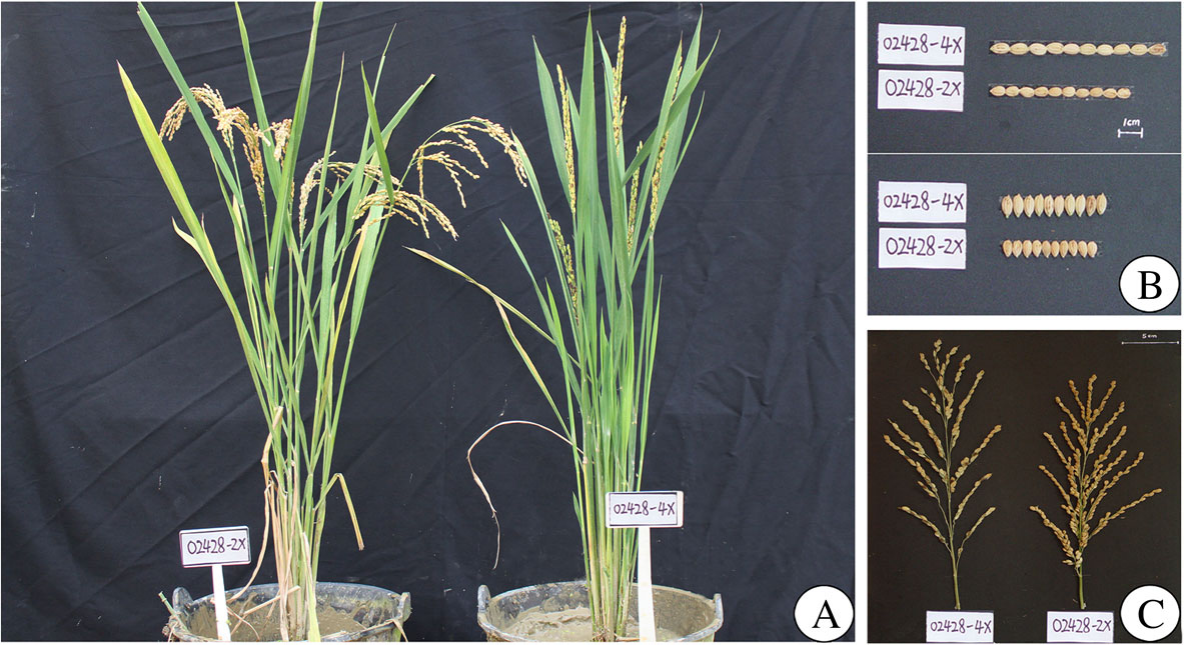
Genetic variations in diploid and autotetraploid rice. (**a**) The diploid (*left*) and autotetraploid (*right*) rice. (**b**) Grain length and width of 02428-2x and 02428-4x. (**c**) Panicles of 02428-2x and 02428-4x

The ploidy levels of autotetraploid rice, 02428-4x, was stable throughout the consecutive generations according to the chromosome observations (Additional file 1: Figure S1). We found significant variations in morphological/agronomic traits of 02428-4x and 02428-2x (Fig. 1; Additional file 2: Table S1). The seed set was significantly lower in 02428-4x than 02428-2x, with a marked difference of 82.47%. 02428-4x also depicted poor pollen fertility (43.3%) and embryo sac fertility (55.48%) (Additional file 2: Table S2), which may be the major reason for low seed set.

**Fig. 2:**
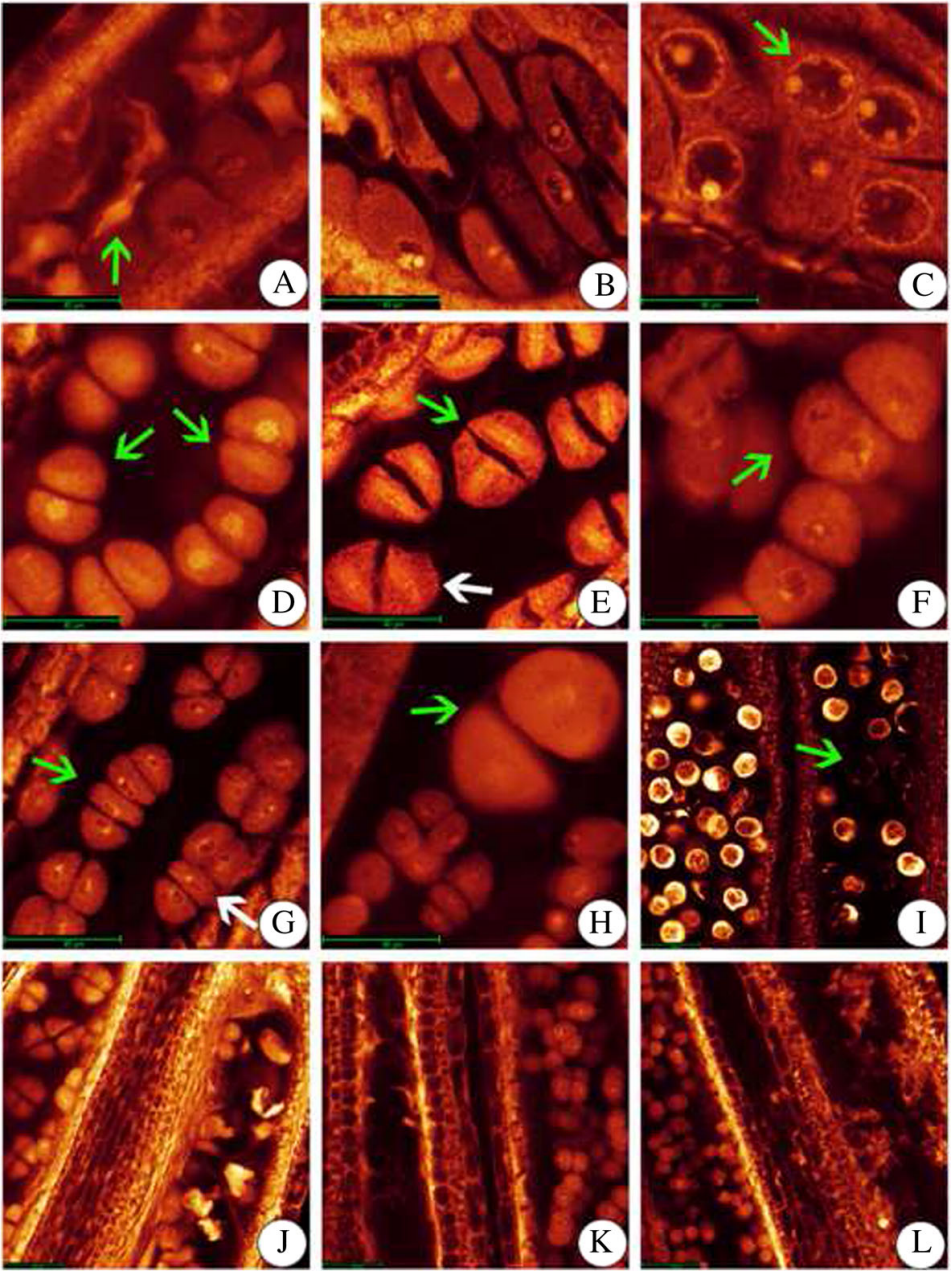
Abnormalities during pollen mother cell (PMC) meiosis in 02428-4x. (**a**, **b**) degeneration of pollen mother cells (*green arrow*). (**c**) multiple nucleoli PMC (*green arrow*). (**d**) asynchronous division of PMC (*green arrow*), with one at metaphase II and another at prophase II. (**e**) abnormal spindle during formation of dyad, including ‘T’ type (*green arrow*) and oblique type (*white arrow*). (**f**) triad (*green arrow*). (**g**) abnormal tetrad, *green arrow* shows the linear type and *white* one shows the ‘T’ type. (**h**) abnormal giant tetrad (*green arrow*). (**i**) abortive pollens in bi-cellular pollen stage, with large and small pollen grains and spherical abortion type (*green arrow*). (**j**-**l**) degeneration of meiocytes in tetrad (**j**, **k**) and early microspore stage (**l**). Bars = 40 μm

WE-CLSM (whole-mount eosin B-staining confocal laser scanning microscopy) observation revealed that the stages of pollen development in autotetraploid rice were greatly in accordance with the sampling standards of diploid rice (Additional file 2: Table S3A). The stages of pollen development were same in 02428-4x and 02428-2x, and they could be divided into eight different stages (Additional file 1: Figure S2). However, many abnormalities (~34%), including degeneration of pollen mother cell (PMC) (Fig. 2a, b, j-l), multiple nucleoli (Fig. 2c), asynchronous division of PMC (Fig. 2d), abnormal spindle in dyad (Fig. 2e), abnormal tetrads (Figs. 2f- h) and abortive pollens at bi-cellular pollen stage (Fig. 2i), were observed in 02428-4x (Additional file 2: Table S4). In addition, abnormal chromosome behavior and chromosomal configurations were found during PMC meiosis of 02428-4x (Additional file 1: Figure S3; Additional file 2: Table S5).

**Fig. 3:**
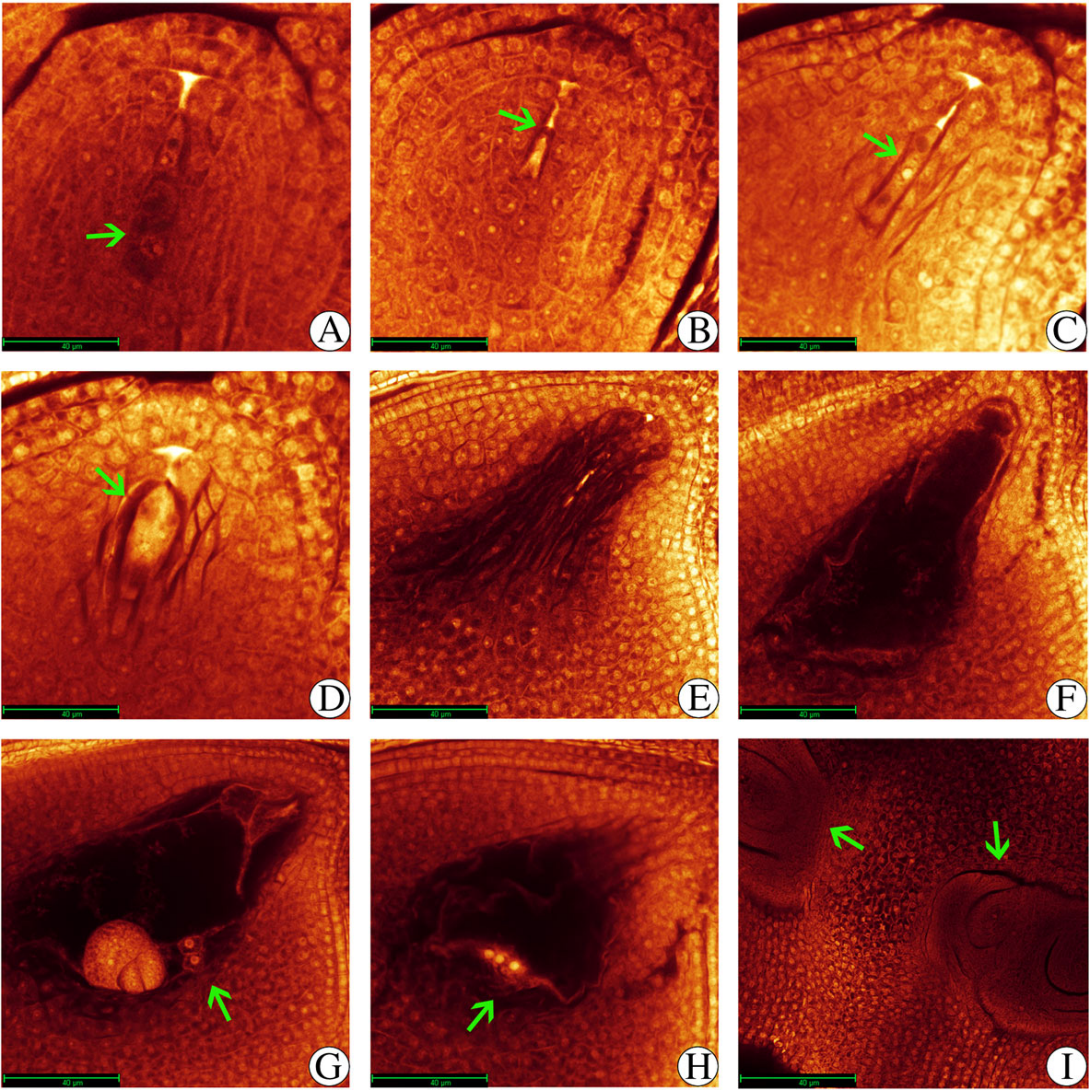
Abnormalities during embryo sac development in 02428-4x. (**a**, **b**) functional megaspores degeneration (*green arrow*). (**c**) abnormal mono-nucleate embryo sac (*green arrow*). (**d**, **e**, **f**) embryo sac degeneration (*green arrow*). (**g**, **h**) abnormal position and number of polar nuclei (*green arrow*). (**i**) double ovules (*green arrow*). Bars = 40 μm

Moreover, WE-CLSM was also employed to observe the embryo sac development from the same spikelet. The embryo sac development stages were synchronous with the pollen development stages in diploid rice (Additional file 2: Table S3B), namely pre-meiotic interphase, meiosis, single microspore stage and bi-cellular pollen stage were corresponding to the megasporocyte formation stage, megasporocyte meiosis stage, functional megaspore formation stage and eight-nucleate embryo sac developing-stage, respectively. Similar to normal diploid rice, embryo sac development in the autotetraploid rice could be divided into eight development stages (Additional file 1: Figure S4). Different kinds of abnormalities were observed during the development of embryo sac in 02428-4x compared to 02428-2x. The main kinds of abnormalities were functional megaspores degeneration (Fig. 3a, b), abnormal mono-nucleate embryo sac (Fig. 3c), embryo sac degeneration (Fig. 3d-f), and abnormal polar nuclei in number and position (Fig. 3g, h). Some double ovules with unsynchronized development were also observed in the autotetraploid rice (Fig. 3i).

### Variations in miRNAs expression profiles during pollen and embryo sac development in autotetraploid rice

A total of eight miRNA libraries of four pollen stages in anther, including pre-meiotic interphase (PMA), meiosis (MA), single microspore stage (SCP) and bi-cellular pollen stage (BCP) and four corresponding stages of embryo sac development in ovary, including megasporocyte formation stage (MF), megasporocyte meiosis stage (MM), functional megaspore formation stage (FMF) and eight-nucleate embryo sac developing-stage (EES), were prepared from autotetraploid and diploid (CK) rice. Principal component analysis showed that all libraries clustered into two groups, which related to pollen or embryo sac development, and each stage can be separated from PMA to BCP/MF to EES (Additional file 1: Figure S5). Moreover, 28 miRNAs were randomly selected for qPCR verification (Additional file 1: Figure S6). The expression patterns of the 28 miRNAs obtained through qPCR were largely consistent with the microRNAomes data. These results demonstrated the accuracy of samples used in the present study.

A total of 748 miRNAs, including 260 known and 488 novel miRNAs, were found in the present study (Additional file 2: Table S6). Of these miRNAs, 572 and 541 expressed during the development of pollen in diploid and autotetraploid rice, while 370 and 486 were associated with the development of embryo sac in diploid and autotetraploid rice, respectively (Additional file 2: Table S7). Among the total miRNAs, 43 miRNAs (29 novel) were specifically detected during the pollen development in autotetraploid rice (12, 1, 1 and 29 in PMA-4x, MA-4x, SCP-4x and BCP-4x, respectively), and 49 miRNAs (47 novel) were specifically related to the development of embryo sac in autotetraploid rice (13, 20, 6 and 10 in MF-4x, MM-4x, FMF-4x and EES-4x, respectively) (Additional file 2: Table S8).

**Fig. 4:**
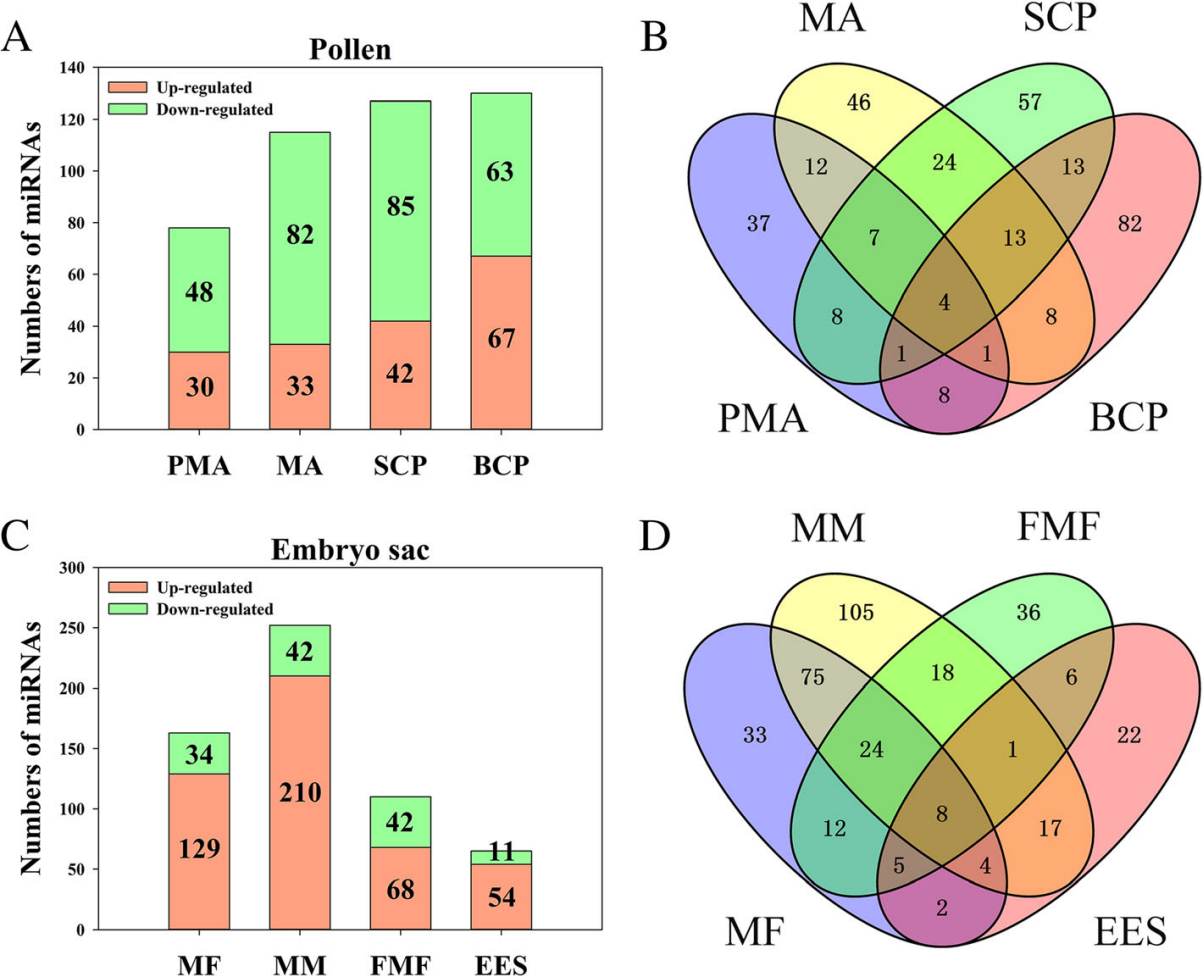
Analysis of DEM (differentially expressed miRNAs) in 02428-4x compared to 02428-2x during pollen and embryo sac development. (**a**, **c**) The number of DEM at different pollen development stages (**a**) and embryo sac development stages (**c**) in 02428-4x; (**b**, **d**) Venn analysis of DEM during pollen (B) and embryo sac (**d**) development in 02428-4x. PMA, MA, SCP and BCP represent pre-meiotic interphase, meiosis and single microspore stage and bi-cellular pollen stage, respectively. MF, MM, FMF and EES represent megasporocyte formation stage, megasporocyte meiosis stage, functional megaspore formation stage and eight-nucleate embryo sac developing-stage, respectively

We detected 321 DEM (Differentially expressed miRNAs) during pollen development in autotetraploid compared to diploid rice, which accounted for ~50% of the total detected miRNAs. Of these DEM, 78 (i.e. 30 up- and 48 down-regulated) were detected in PMA, 115 (i.e. 33 up- and 82 down-regulated) in MA, 127 (i.e. 42 up- and 85 down-regulated) in SCP and 130 miRNAs (i.e. 67 up- and 63 down-regulated) in BCP of autotetraploid compared to diploid rice (Fig. 4a; Table 1). Three DEM, including *PC-3p-199234_121*, *PC-3p-353219_42* and *PC-3p-46519_401*, exhibited co-down-regulation in four pollen development stages, and *osa-miR2275d* were found to be down-regulated in PMA, MA and SCP but up-regulated in BCP (Fig. 4b; Additional file 3: Table S9).

**Table 1:** Differentially expressed miRNAs (DEM) in autotetraploid rice compared to diploid rice

Comparison	Up-regulated	Down-regulated	Total	Targets	Gene Ontology
PMA-4x/2x	30	48	78	470	GO: 0005576 (extracellular region)
MA-4x/2x	33	82	115	664	GO: 0007275 (multicellular organismal development) GO: 0005576 (extracellular region)
SCP-4x/2x	42	85	127	504	GO: 0006139 (nucleobase, nucleoside, nucleotide and nucleic acid metabolic process) GO: 0009653 (anatomical structure morphogenesis) GO: 0030154 (cell differentiation) GO: 0007165 (signal transduction) GO: 0009908 (flower development) GO: 0003700 (transcription factor activity)
BCP-4x/2x	67	63	130	548	GO: 0009908 (flower development) GO: 0007165 (signal transduction) GO: 0009653 (anatomical structure morphogenesis) GO: 0006139 (nucleobase, nucleoside, nucleotide and nucleic acid metabolic process) GO: 0003700 (transcription factor activity) GO: 0003723 (RNA binding)
MF-4x/2x	129	34	163	489	GO: 0009908 (flower development) GO: 0009653 (anatomical structure morphogenesis) GO: 0006139 (nucleobase, nucleoside, nucleotide and nucleic acid metabolic process) GO: 0007165 (signal transduction) GO: 0030154 (cell differentiation) GO: 0003700 (transcription factor activity) GO: 0016301 (kinase activity)
MM-4x/2x	210	42	252	835	GO: 0044238 (primary metabolic process) GO: 0007165 (signal transduction) GO: 0000166 (nucleotide binding) GO: 0016301 (kinase activity) GO: 0003700 (transcription factor activity)
FMF-4x/2x	68	42	110	432	GO: 0009856 (pollination) GO: 0009653 (anatomical structure morphogenesis) GO: 0005488 (binding)
EES-4x/2x	54	11	65	285	GO: 0003700 (transcription factor activity)

PMA, MA, SCP and BCP represent pre-meiotic interphase, meiosis and single microspore stage and bi-cellular pollen stage, respectively. MF, MM, FMF and EES represent megasporocyte formation stage, megasporocyte meiosis stage, functional megaspore formation stage and eight-nucleate embryo sac developing-stage, respectively. “4x” and “2x” represent the autotetraploid and diploid rice, respectively

In total, 368 DEM were found during embryo sac development of autotetraploid rice compared to diploid rice (Fig. 4c; Table 1), including 163 (129 up- and 34 down-regulated) in MF, 252 (210 up- and 42 down-regulated) in MM, 110 (68 up- and 42 down-regulated) in FMF and 65 (54 up- and 11 down-regulated) in EES. Interestingly, higher numbers of down-regulated miRNAs were detected during pollen development, while up-regulated miRNAs were more commonly found during embryo sac development. Similar phenomenon was observed by Venn analysis during embryo sac development, and the results showed that there was a great variation in number of DEM during different embryo sac development stages, and the highest number of DEM (105) was observed during MM (Fig. 4d; Additional file 3: Table S10). Venn analysis revealed that only eight miRNAs were co-up-regulated in each stage compared to diploid rice, including *osa-miR1423-5p_L-2R + 2*, *osa-miR1432-5p_R + 1*, *osa-miR1862d*, *osa-miR2094-3p_L-1R + 3*, *osa-miR5150-3p*, *osa-miR535-3p*, *PC-3p-115563_179* and *PC-3p-46519_401* (Fig. 4d; Additional file 3: Table S10).

We further compared the DEM between pollen and embryo sac development in each corresponding stage (Additional file 1: Figure S7; Additional file 3: Table S11). In the first comparison (PMA/MF), we found four DEM co-up-regulated and one DEM co-down-regulated in PMA and MF of autotetraploid rice. 18 DEM were found to be down-regulated in PMA of autotetraploid rice, but up-regulated in MF. In the second comparison (MA/MM), two DEM showed co-up-regulation and two DEM exhibited co-down-regulation during MA and MM in autotetraploid rice, respectively. 56 DEM showed opposite trends in autotetraploid rice, which were up-regulated in MA but down-regulated in MM (15) or down-regulated in MA but up-regulated in MM (41). In the third comparison (SCP/FMF), nine DEM displayed co-up-regulation in SCP and FMF of autotetraploid rice, but no co-down-regulated DEM was observed. Five DEM were up-regulated in SCP but down-regulated in FMF and nine DEM were down-regulated in SCP but up-regulated in FMF. In the fourth comparison (BCP/EES), six co-up-regulated DEM and three co-down-regulated DEM were found in BCP and EES of autotetraploid rice, and six DEM showed down-regulation in BCP, while up-regulation in EES. Interestingly, higher number of DEM was found in each corresponding pollen and embryo sac development stage than co-regulated DEM (Additional file 1: Figure S7; Additional file 3: Table S11). Furthermore, we compared the detected miRNAs between pollen and embryo sac development in autotetraploid rice. A total of 617 miRNAs showed differential expression levels during the embryo sac development compared to pollen development (Additional file 1: Figure S8A; Additional file 3: Table S12), including 384 (i.e. 186 up- and 198 down-regulated), 400 (i.e. 236 up-and 164 down-regulated), 250 (i.e. 101 up- and 149 down-regulated) and 300 DEM (i.e. 77 up- and 223 down-regulated) in MF, MM, FMF and EES, respectively. Interestingly, 111 DEM were co-expressed during all pollen and embryo sac development stages (Additional file 1: Figure S8B), including 22 co-up- and 58 co-down-regulated during embryo sac development stages in autotetraploid rice, while these DEM showed totally different expression levels during pollen development stages (i.e. 22 co-down- and 58 co-up-regulated). Therefore, we defined these DEM as embryo sac-enriched miRNAs (22), which was designated as DEM-ES, and pollen-enriched miRNAs (58), which was designated as DEM-P, in autotetraploid rice (Additional file 1: Figure S8C; Additional file 3: Table S13).

### Target prediction of DEM and functional classification of pollen and embryo sac mother cell meiosis-related targets

To understand the genetic variations in autotetraploid rice during the pollen and embryo sac development, we predicted targets of 748 miRNAs using the Targetfinder, and 2260 targets were found, with an average of 3.0 targets per miRNA (Additional file 3: Table S14). Gene Ontology (GO) enrichment analyses showed that DEM-P were significantly involved in flower development (GO: 0009908) and transcription factor activity (GO: 0003700), while cell differentiation (GO: 0030154), cell death (GO: 0008219), signal transduction (GO: 0007165) and nucleus (GO: 0005634) terms were detected by DEM-ES of autotetraploid rice (Additional file 1: Figures S9, S10). GO analysis of the targets of DEM at each stage in pollen and embryo sac development is shown in Table 1. The predicted targets were enriched in multicellular organismal development (GO: 0007275) and extracellular region (GO: 0005576) in MA during pollen development (Additional file 1: Figure S11). However, primary metabolic process (GO: 0044238), signal transduction (GO: 0007165), nucleotide binding (GO: 0000166), kinase activity (GO: 0016301) and transcription factor activity (GO: 0003700) were preferentially enriched in MM during embryo sac development (Additional file 1: Figure S12).

Meiosis is a crucial process during pollen development. We analyzed the DEM related to the meiosis of pollen development, and compared the target genes predicted by the DEM at MA in autotetraploid rice with the transcriptome data reported for meiosis-related and stage-specific gene expressions in *Arabidopsis*, diploid and autotetraploid rice [6, 29–37]. By using the predicted targets of the up-regulated miRNAs in MA, we identified 18 DEM associated with meiosis (Additional file 3: Table S15). Two meiosis-related genes (i.e. *LOC_Os02g48010* predicted by *osa-MIR5083-p3* that encoded nuclear matrix constituent protein 1-like and *LOC_Os02g42230* predicted by *osa-MIR159a-p5* that encoded RPA2B - Putative single-stranded DNA binding complex subunit 2), and three meiosis specific genes that showed down-regulation in autotetraploid compared to the diploid rice during meiosis, including *LOC_Os09g32020* (predicted by *osa-MIR159a-p5*), *LOC_Os06g40550* (predicted by *osa-miR5504_R-3*), and *LOC_Os04g21590* (predicted by *osa-miR5504_R-3*), which annotated as ubiquitin fusion degradation protein, ABC-2 type transporter domain containing protein and PB1 domain containing protein, respectively. Another important gene, *LOC_Os01g59660* (*OsGAMYB*, predicted by *osa-miR159a.1*), which is required for pollen development and encodes MYB family transcription factor, was also detected. Some important genes, such as *LOC_Os07g36940* predicted by the *osa-miR5836*, and *LOC_Os01g47530* predicted by the *osa-miR159a.1*, were found to be down-regulated during meiosis stage (Additional file 3: Table S15). Moreover, 14 genes showed differential expression patterns in transcriptome sequencing, and the targets of up-regulated DEM were similar to these 14 genes, such as *LOC_Os03g63390* (predicted by *osa-miR528-5p*) annotated as plastocyanin-like domain containing protein, *LOC_Os08g23880* (predicted by *gma-miR6300_R + 3*) annotated as no apical meristem (NAM) protein and *LOC_Os10g30150* (predicted by *osa-miR3979-5p_R + 1*) annotated as universal stress protein domain containing protein (Additional file 3: Table S15). All the aforementioned targets may relate to abnormal meiosis of PMC.

The development process of embryo sac was different from pollen, especially after meiosis of the embryo sac mother cell (EMC); therefore, the regulation mechanism of embryo sac development was also different from that in pollen. We compared the target genes predicted by the DEM in MM associated with the embryo sac development in autotetraploid rice with the previously reported transcriptome data for megagametogenesis-related and stage-specific expression in diploid rice and *Arabidopsis* [8, 9, 38–40], and 21 DEM were found to be related to the development of embryo sac mother cell (Additional file 3: Table S16). Among the up-regulated miRNAs of MM in autotetraploid, 26 predicted targets were associated with the megasporocyte meiosis stage. Two genes, *LOC_Os06g47830* (*RPA2C,* predicted by *osa-miR167h-3p*) and *LOC_Os12g20324* (*CYCA1;3*, predicted by *PC-5p-896126_17*) were associated with meiosis. *LOC_Os06g11500* and *LOC_Os06g48060* (predicted by *osa-miR1436_L + 3_1ss5CT*, a specific DEM in MM), annotated as minichromosome maintenance (MCM) family subunit 9 and ABC transporter, were highly expressed in zygotene and pachytene of EMC. Nine genes showed the highest expression in tetrad, such as *LOC_Os12g42160*, which encoded kinesin motor domain containing protein (predicted by the MM specific DEM, *PC-5p-983436_13* and *osa-miR1436_L + 3_1ss5CT*) and *LOC_Os06g48030* (predicted by the MM specific DEM, *osa-MIR814a-p3_1ss24AT* and *PC-5p-430417_41*) (Additional file 3: Table S16).

Moreover, we used the targets of the DEM in MA and MM to analyze protein-protein interaction networks. Of these targets, eight genes associated with meiosis exhibited interactions with 39 targets, including *LOC_Os09g32020* (*OsTKPR1*, ubiquitin fusion degradation protein), *LOC_Os08g40440* (dihydroflavonol-4-reductase) and *LOC_Os06g40550* (ABC-2 type transporter domain containing protein), identified by Fujita et al. [29], *LOC_Os05g51790* (SMC5, RecF/RecN/SMC N terminal domain containing protein) and *LOC_Os02g42230* (RPA2B - Putative single-stranded DNA binding complex subunit 2) identified by Jin et al. [31], *LOC_Os01g59660* (*OsGAMYB*, MYB family transcription factor) identified by Aya et al. [32], *LOC_Os06g47830* (RPA2C - Putative single-stranded DNA binding complex subunit 2) identified by Wang et al. [40] and *LOC_Os12g20324* (CYCA1;3) found in oryzabase (http://www.shigen.nig.ac.jp/rice/oryzabase/) (Additional file 1: Figure S13; Additional file 4: Table S17). The DEM detected in MA during pollen development was different from the embryo sac development, and these DEM showed divergent expression patterns. *OsGAMYB* encoded by *LOC_Os01g59660* (predicted by *osa-miR159a.1*, which showed up-regulation in male meiosis but down-regulation in female meiosis), revealed interaction with the genes encoding splicing factor 3B subunit 1 (*LOC_Os02g05410* and *LOC_Os02g05310* predicted by *osa-miR5504_R-3*). CYCA1;3 (*LOC_Os12g20324* predicted by *PC-5p-896126_17*, which showed up-regulation in female meiosis) interacted with the genes encoding MYB family transcription factor and putative minichromosome maintenance (MCM) family subunit 9 (*LOC_Os06g11500* predicted by *osa-miR1436_L + 3_1ss5CT*, which displayed down-regulation in male meiosis but up-regulation in female meiosis). RPA2C (*LOC_Os06g47830* predicted by *osa-miR167h-3p*) and RPA2B (*LOC_Os02g42230* predicted by *osa-MIR159a-p5*, which found to be up-regulated in male meiosis but down-regulated in female meiosis) encoded replication proteins, A2C and A2B, and showed interaction with *LOC_Os06g11500*, which further interacted with CYCA1;3. *OsTKPR1* (*LOC_Os09g32020* encoded ubiquitin fusion degradation protein), which was another target predicted by *osa-MIR159a-p5*, interacted with the mov34 family protein (*LOC_Os05g46490* predicted by *osa-miR1436_L + 3_1ss5CT*), Sel1 repeat domain containing protein (*LOC_Os03g15350* predicted by *osa-miR1436_L + 3_1ss5CT*) and DnaK family protein genes (*LOC_Os12g14070*, predicted by *osa-miR3979-5p_R + 1*, which showed up-regulation in male meiosis, and *LOC_Os02g53420* and *LOC_Os03g02260*, predicted by *osa-miR164e_R-3*, which displayed down-regulation in female meiosis). The SMC5 gene encoded RecF/RecN/SMC N terminal domain containing protein (*LOC_Os05g51790* predicted by *osa-miR1861h*), which interacted with *LOC_Os05g05440* (annotated as expressed protein and predicted by *osa-MIR5083-p3*, which was found to be up-regulated in male meiosis but down-regulated in female meiosis) and *LOC_Os08g37444* (annotated as signal recognition particle receptor and predicted by *hvu-miR1436_L + 3_2ss5CT13GA*, which exhibited up-regulation in female meiosis). In addition, *LOC_Os08g40440* (predicted by *osa-miR1436_L + 3_1ss5CT*) encoded dihydroflavonol-4-reductase that interacted with ABC-2 type transporter domain containing protein (*LOC_Os06g40550* predicted by *osa-miR164e_R-3*, *osa-miR5504_R-3*) and FG-GAP repeat-containing protein (*LOC_Os03g61050* predicted by *osa-miR812v_1ss4GA* that up-regulated in female meiosis). Overall, complex protein-protein interaction networks were detected by the meiosis-related gene and meiosis-related DEM.

### PhasiRNAs accumulation during the pollen and embryo sac development in autotetraploid rice

**Fig. 5:**
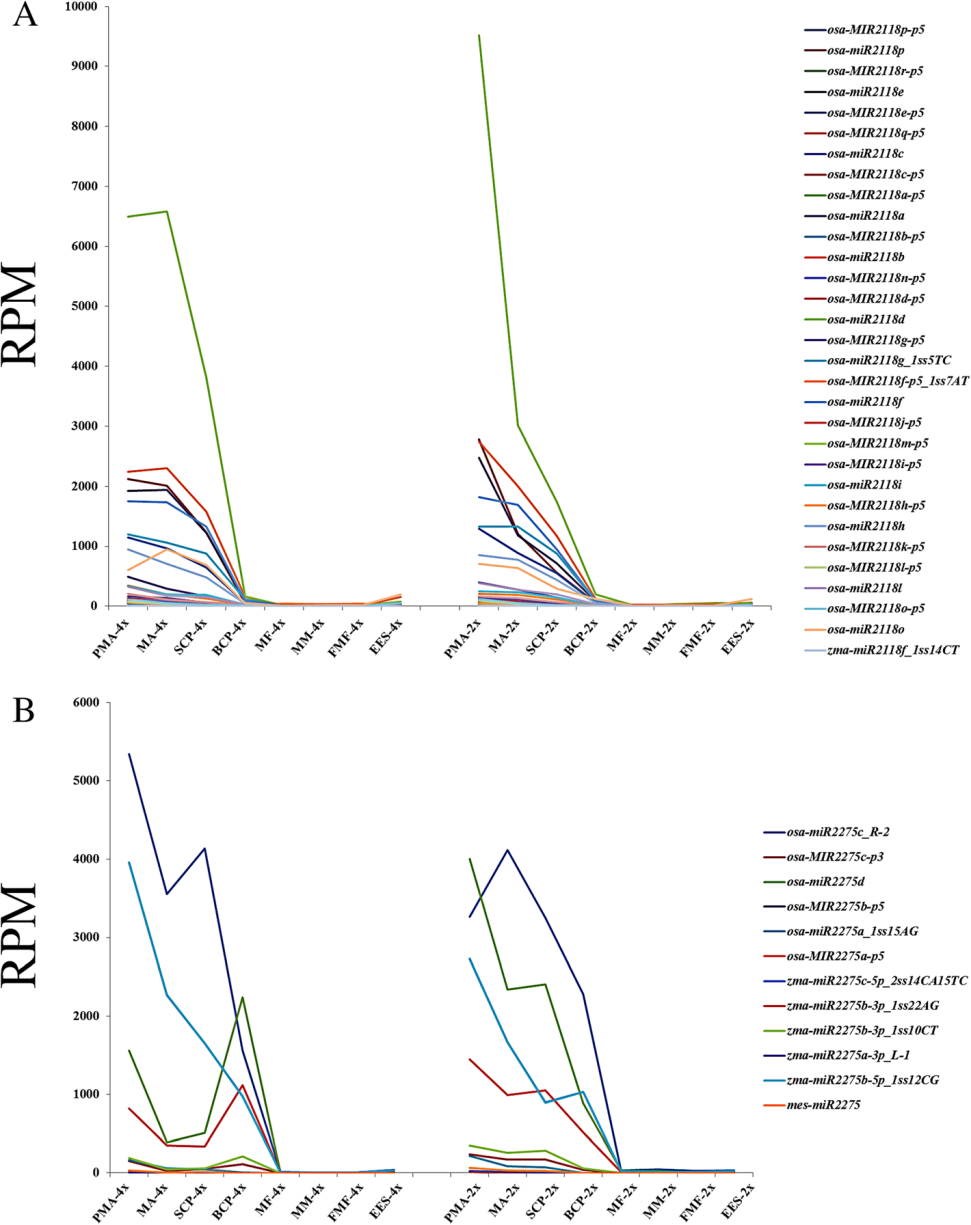
The expression patterns of *miR2118* (**a**) and *miR2275* (**b**) families between 02428-4x and 02428-2x. PMA, MA, SCP and BCP represent pre-meiotic interphase, meiosis and single microspore stage and bi-cellular pollen stage, respectively. MF, MM, FMF and EES represent megasporocyte formation stage, megasporocyte meiosis stage, functional megaspore formation stage and eight-nucleate embryo sac developing-stage, respectively. “4x” and “2x” represent the autotetraploid and diploid rice, respectively

The expression patterns of *miR2118* and *miR2275* families from PMA to BCP in pollen and from MF to EES in embryo sac were nearly consistent in 02428-4x and 02428-2x, which specifically enriched during pollen development and gradually decreased after PMA (Fig. 5). Six members of *miR2118* family showed differential expression patterns during pollen development (Table 2). Two of them, *osa-miR2118d* and *osa-MIR2118e-p5*, were found to be up-regulated in MA. Beside, six members of *miR2275* also exhibited differential expression patterns in 02428-4x, including *osa-miR2275d*, *osa-MIR2275c-p3*, *mes-miR2275*, *zma-miR2275a-3p_L-1*, *zma-miR2275b-3p_1ss10CT* and *zma-miR2275b-3p_1ss22AG* (Table 2). Interestingly, all these six miRNAs of *miR2275* family were found to be down-regulated in MA of 02428-4x.

**Table 2:** The regulation of *miR2118* and *miR2275* families during pollen development of autotetraploid rice

miRNA Family	miRNA name	PMA	MA	SCP	BCP
*miR2118*	*osa-miR2118d*		up	up	
	*osa-miR2118p*			up	
	*osa-miR2118o*			up	down
	*osa-miR2118i*				up
	*osa-MIR2118i-p5*			up	
	*osa-MIR2118e-p5*		up		
*miR2275*	*osa-miR2275d*	down	down	down	up
	*mes-miR2275*	down	down	down	
	*osa-MIR2275c-p3*		down	down	up
	*zma-miR2275b-3p_1ss10CT*		down	down	up
	*zma-miR2275b-3p_1ss22AG*		down	down	up
	*zma-miR2275a-3p_L-1*	down	down		

Up: up-regulated in autotetraploid rice; Down: down-regulated in autotetraploid rice; PMA, MA, SCP and BCP represent pre-meiotic interphase, meiosis and single microspore stage and bi-cellular pollen stage, respectively

**Fig. 6:**
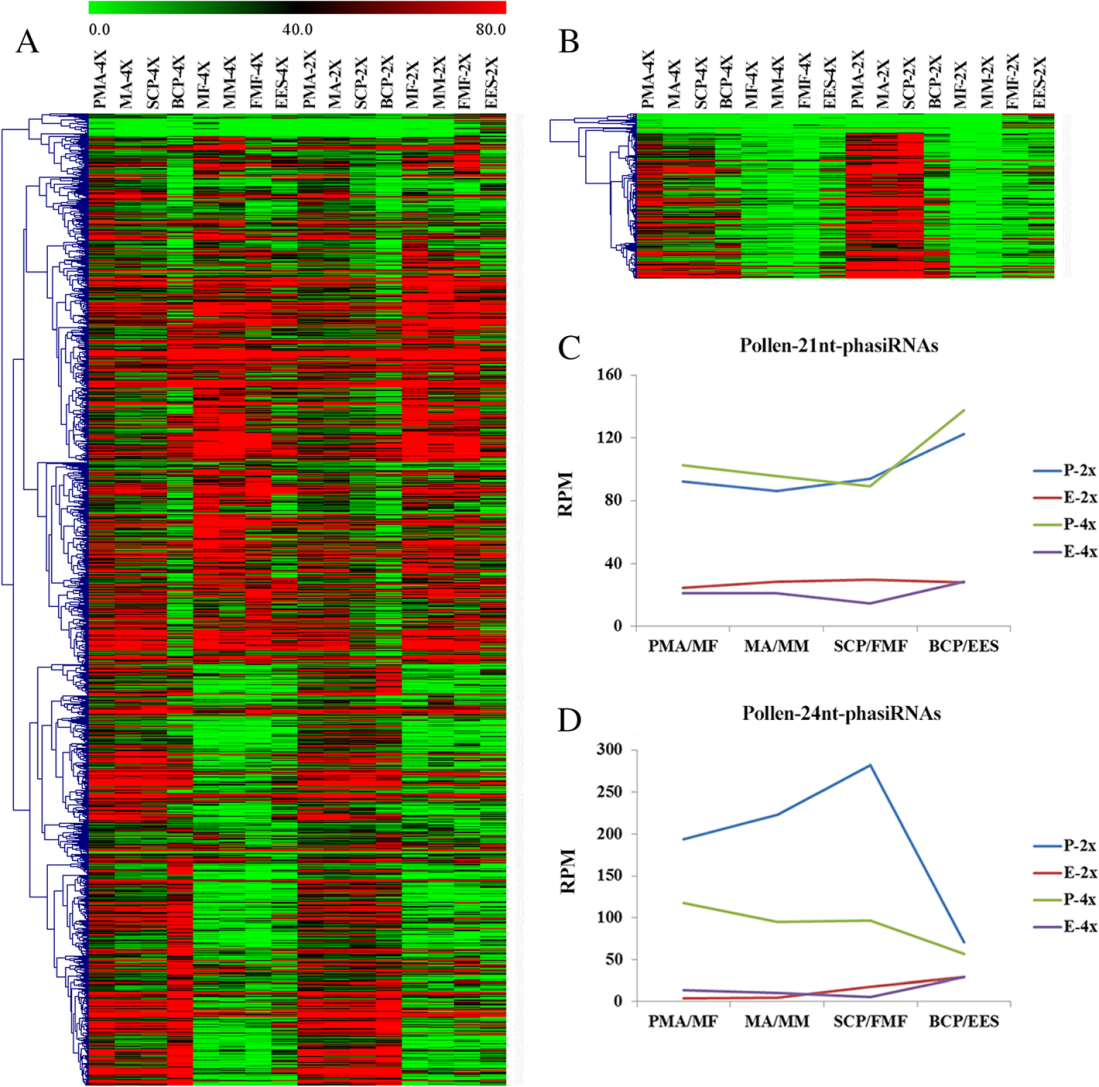
The distribution of 21 nt- and 24-phasiRNAs in auotetraploid and diploid rice. (**a**, **b**) Hierarchical cluster analysis of 21 nt-phasiRNAs (**a**) and 24-phasiRNAs (**b**) in different libraries was constructed by MultiExperiment View (version 4.9). The scale bar indicates the RPM of phasiRNAs; (**c**, **d**) Abundance of 21 nt-phasiRNAs and 24 nt-phasiRNAs in different libraries. P-4x and P-2x indicate autotetraploid and diploid pollen development. E-4x and E-2x indicate autotetraploid and diploid embryo sac development, respectively

Based on PhaseTank analysis [41], we observed 1117 loci generating 21 nt-phasiRNAs and 190 loci generating 24 nt-phasiRNAs triggered by the *miR2118* and *miR2275*, respectively (Additional file 4: Tables S18, S19). We found that 21 nt-phasiRNAs were not only detected during the development of pollen, but also during the development of embryo sac, while 24 nt-phasiRNAs only accumulated during the pollen development (Figs. 6a, b). By Z-score transformation [34], 451 21 nt-phasiRNAs and 179 24 nt-phasiRNAs were preferentially expressed during the pollen development. Pollen-specific-21 nt-phasiRNAs were accumulated from PMA and retained the expression levels in BCP of 02428-2x, and similar trend was detected in 02428-4x (Fig. 6c). In contrast, pollen-specific-24 nt-phasiRNAs appeared at PMA and reached to the highest level in SCP, and then declined in BCP of 02428-2x, whereas gradual decline was observed in 02428-4x (Fig. 6d). Furthermore, only 109 out of 451 pollen-specific-21 nt-phasiRNAs showed different levels of expression between 02428-4x and 02428-2x. Up-regulated pollen-specific-21 nt-phasiRNAs were dominant in PMA and BCP, but more number of down-regulated pollen-specific-21 nt-phasiRNAs was detected in MA and SCP (Table 3). The expression levels of *miR2118* (i.e. *osa-miR2118d*, *osa-miR2118p*, *osa-miR2118o*, *osa-miR2118i*) and their pollen-specific-21 nt-phasiRNAs were quite different during pollen development stages (Tables 2 and 3). A total of 173 out of 179 pollen-specific-24 nt-phasiRNAs showed differential expression patterns in 02428-4x compared to 02428-2x. In addition, almost all of the differentially expressed pollen-specific-24 nt-phasiRNAs were found to be down-regulated during pollen development in autotetraploid rice, especially at MA and SCP, which were in accordance with the spatial-temporal expressions of *miR2275* (i.e. *osa-miR2275d*, which was down-regulated from PMA to SCP and up-regulated in BCP) (Tables 2 and 3).

**Table 3:** Differentially expressed phasiRNAs during pollen development in autotetraploid rice

	PMA		MA		SCP		BCP	
	Up	Down	Up	Down	Up	Down	Up	Down
21 nt-phasiRNAs	18	2	30	50	35	98	79	22
*osa-miR2118d*	4	0	6	20	5	25	23	5
*osa-miR2118p*	4	0	6	4	6	11	13	1
*osa-miR2118o*	6	0	7	12	7	26	21	5
*osa-miR2118i*	1	0	1	2	1	8	5	2
24 nt-phasiRNAs	6	38	0	138	0	153	22	13
*osa-miR2275d*	2	14	0	54	0	63	9	5

Up: up-regulated in autotetraploid rice; Down: down-regulated in autotetraploid rice; PMA, MA, SCP and BCP represent pre-meiotic interphase, meiosis and single microspore stage and bi-cellular pollen stage, respectively. *osa-miR2118d*, *osa-miR2118p*, *osa-miR2118o* and *osa-miR2118i* indicate the 21 nt-phasiRNAs triggered by them; *osa-miR2275d* indicates the 24 nt-phasiRNAs triggered by it

### siRNAs, associated with transposable elements, showed differential expression patterns in autotetraploid rice

**Fig. 7:**
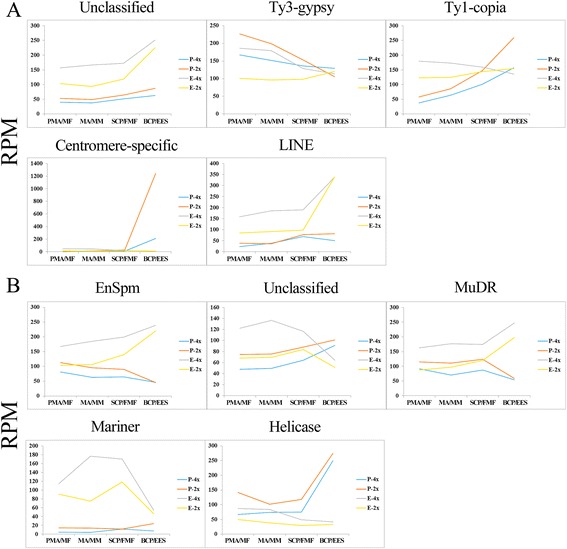
Abundance of TEs-siRNAs (siRNAs associated with transposable elements) in 02428-4x and 02428-2x during pollen and embryo sac development. (**a**) 24 nt TEs-siRNAs in class I (retrotransposons). (**b**) 24 nt TEs-siRNAs in class II (transposon). PMA, MA, SCP and BCP represent pre-meiotic interphase, meiosis and single microspore stage and bi-cellular pollen stage, respectively. MF, MM, FMF and EES represent megasporocyte formation stage, megasporocyte meiosis stage, functional megaspore formation stage and eight-nucleate embryo sac developing-stage. P-4x and P-2x indicate autotetraploid and diploid pollen development, respectively. E-4x and E-2x indicate autotetraploid and diploid embryo sac development, respectively

We documented 4386 siRNAs associated with transposable elements (TEs-siRNAs) during pollen and embryo sac development of 02428-4x and 02428-2x. 24-nt TEs-siRNAs, 3106 of 4386 siRNAs, occupied the great majority of the siRNA population (Additional file 4: Table S20). Of 3106 TEs-siRNAs (609 associated with pollen development library and 2734 associated with embryo sac development library), 372 and 2497 were specifically associated with pollen and embryo sac development, respectively, and 237 TEs-siRNAs were expressed during both pollen and embryo sac development (Additional file 1: Figure S14). In contrast to the pollen development, the 24 nt TEs-siRNAs were abundant in embryo sac development. We classified these 24 nt TEs-siRNAs in class I (retrotransposons) with five types and class II (transposon) with eight types (Additional file 4: Table S21). 24 nt TEs-siRNAs showed different expression patterns during the pollen and embryo sac development in 02428-4x, as well as in the 02428-2x; however, some changes were found during the pollen and embryo sac development when compared to each other (Fig. 7). Compared to the diploid rice, 24 nt TEs-siRNAs were abundantly expressed during embryo sac development of 02428-4x, whereas low levels of expressions were found in pollen development of 02428-4x (Fig. 7). Of these 24 nt TEs-siRNAs, 59% (584/988) of MF, 69% (1152/1672) of MM, 49% (524/1066) of FMF and 25% (475/1877) of EES were differentially expressed during embryo sac development of 02428-4x, and 39% (113/287) of PMA, 50% (148/296) of MA, 57% (179/312) of SCP and 56% (115/206) of BCP were differentially expressed during pollen development (Additional file 4: Table S22). Most of the differentially expressed TEs-siRNAs (96%, 96%, 92% and 85% in MF, MM, FMF and EES, respectively) were up-regulated in embryo sac development of 02428-4x. Particularly, the MM stage exhibited the highest numbers (1106) of differentially expressed TEs-siRNAs. On the other hand, higher number of down-regulated TEs-siRNAs (81%, 84%, 79% and 88% in PMA, MA, SCP and BCP) were identified in 02428-4x than 20428-2x during the pollen development (Additional file 4: Table S22).

## Discussion

### Polyploidy associated miRNAs expression patterns during pollen and embryo sac development in autotetraploid rice

Transcriptome analysis of rice ovules and anthers revealed that only 448 genes representing ovule-exclusive genes, compared to 1149 anther-exclusive genes, and different GO terms were found in ovules and anthers [8]. Here, large numbers of miRNAs were detected in pollen and embryo sac library and spatiotemporally dynamics during the pollen and embryo sac development. We found some miRNAs were exclusive during the development of pollen or embryo sac and some were exclusive in autotetraploid/diploid rice; suggesting that miRNAs expressed during pollen development were different from embryo sac development, and some miRNAs changed after polyploidization. MircoRNAome analysis of different species of diploid and autotetraploid *Paulownia* showed that up-regulated miRNAs were more common than down-regulated miRNAs in autotetraploid *P. tomentosa*, *P. fortunei*, and *P. australis* except in autotetraploid *P. ‘Yuza 1’* [42–45]. In our study, the number of down-regulated DEM was higher in pollen development of autotetraploid than diploid rice, whereas Li et al. [10] reported higher number of up-regulated DEM in pollen development of another autotetraploid line than diploid counterpart. Beside, differences between the pollen and embryo sac development in 02428-4x, up-regulated DEM were enriched during embryo sac development, particularly at the meiosis stage. These studies on polyploid plants suggest greater diversity in miRNAs expression patterns in different species. Polyploidization always cause increase/decrease in expression patterns, gene loss and gene recombination in many plant species [46–49]. Higher missing rate of markers were detected in Alfalfa [50], great variations were found in miRNAs [51, 52], and some differentially expressed miRNAs were found to be associated with different ploidy rice [53]. Here, multitudinous of exclusively DEM detected in each stage during pollen and embryo sac development in autotetraploid rice, and similar phenomena was found in Taichung65-4x [10], where miRNA expression patterns changed dynamically in autotetraploid rice. These studies demonstrated huge changes in miRNAs after polyploidization and polyploidy displayed the major impact on the miRNAs expression pattern. Taken together, a great diversity in the DEM was exclusively documented in autotetraploid rice, and some co-expressed DEM exhibited ploidy-related expression patterns, such as *osa-miR2275d*, probably played a key role, which may be associated with the pollen and embryo sac sterility in autotetraploid rice.

### The contrasting expression patterns of meiosis-related DEM during pollen and embryo sac development in autotetraploid rice

Transcriptome profiling is helpful tool for investigating the molecular characters of male and female gametophytes development in *Arabidopsis* [35, 39]. Genome-wide gene expression profiles of ovules and anthers revealed that dynamic gene expressions play crucial roles during female and male gametogenesis in rice [8, 9, 29, 30]. In the present work, the anthers/ovaries containing the developing male/female gametocytes at specific stages were observed, and identified the targets of DEM associated with meiosis, which were similar to the genes detected by previous studies [8, 9, 29–33]. A total of 18 and 21 DEM associated with meiosis were detected in male and female gametophytes, respectively, and these DEM showed huge differences between male and female meiosis, and probably played important roles during pollen and embryo sac development in autotetraploid rice. The rest of the DEM might be involved in various distinct processes that may also related to reproductive abnormalities in autotetraploid rice. Moreover, some meiosis-related genes were highly expressed in meiosis of both male and female meiocytes, while some showed reverse expression patterns [8]. We identified some special DEM associated with meiosis/meiosis-related genes by protein-protein interaction networks. All of these interactions suggest that the DEM of MA and MM had a significant impact on the expressions of meiosis related or stage-specific genes. Additionally, these DEM showed opposite regulation between pollen and embryo sac, suggesting unsynchronized expression profiles in male and female gametophytes of autotetraploid rice. The regulation mechanisms of meiosis-related DEM were quite different between male and female gametophytes.

Earlier studies have shown that overexpression of *miR159* inhibited mRNA levels of MYB family transcription factors leading to male sterility in *Arabidopsis* [20], and impairing the anthers development in rice [54]. In our study, *osa-miR159a.1* targeted MYB family transcription factor (*OsGAMYB*, responsible for defects in tapetal programmed cell death), which interacted with CYCA1;3, and displayed significant up-regulation in male meiosis but down-regulation in female meiosis. The increased expression of *osa-miR159* during pollen development was consistent with a previous study on pollen sterile line [17]. We supposed that overexpression of *osa-miR159a.1* in meiosis not only repressed the expression of MYB family transcription factor, but also affected the meiosis-related genes, which cause meiosis abnormalities and hampered normal pollen development in autotetraploid rice. In addition, *osa-MIR159a-p5*, another member of *osa-miR159*, targets *LOC_Os09g32020* (ubiquitin fusion degradation protein, *OsTKPR1*), which displayed significant up-regulation in MA of autotetraploid rice. Ubiquitin fusion degradation protein families were important in the process of meiosis, and *OsTKPR1* had reported to be associated with male sterility and pollen wall assembly [55, 56]. Highly-expressed *osa-MIR159a-p5* in MA may partially repressed the expression of *LOC_Os09g32020* and cause meiosis abnormalities in 02428-4x.

*osa-miR1436_L + 3_1ss5CT*, targeted MCM9, which up-regulated in female meiosis of autotetraploid rice but down-regulated in male meiosis. Protein-protein interaction showed that MCM9 have a relationship with RPA2C (predicted by *osa-miR167h-3p*) and RPA2B, the genes related to meiosis. Replication protein A (RPA) is a single-stranded DNA binding protein that plays key role in DNA metabolic pathways. Incomplete meiotic chromosome fragmentation and synapsis were found in *rpa1* mutant suggesting an essential role of RPA1 in DNA replication [57]. Additionally, recent research demonstrated a rice gene, *OsMSH4/5* (*Os07g0486000*), which interacted with RPA complex and required for crossover formation during male and female gametes meiosis [40]. Here, up-regulation of *osa-miR1436_L + 3_1ss5CT* and *osa-miR167h-3p* in female meiosis might be negatively correlated with the expression of MCM9 and RPA2C, which displayed interaction with DNA replication genes, demonstrating that it may cause female meiosis abnormalities in autotetraploid rice.

### Down-regulation of 24 nt-phasiRNAs may cause abnormalities during meiosis in autotetraploid rice

*miR2118* and *miR2275* are required as triggers to initiate the 21 nt and 24 nt-phasiRNAs biogenesis, and have been reported in rice and maize anther development [58–60]. MEL1, rice Argonaute protein, selectively binds the 21nt-phasiRNAs to play an important role in meiosis and male fertility [25]. Recently, Zhai et al. [26] analyzed the phasiRNAs of maize anther, and indicated that epidermis was necessary for producing 21 nt pre-meiotic phasiRNA, whereas tapetal layer was required for 24 nt meiotic phasiRNAs biogenesis; both of these phasiRNAs finally transport to meiotic cells and play their role in meiosis. Our previous study depicted different levels of expression of *miR2118* and *miR2275* families in Taichung65 [10]. In the present study, we found that *miR2118* and *miR2275* accumulated in pollen development and gradually declined from pre-meiotic, and 21 nt-phasiRNAs and 24 nt-phasiRNAs were also detected in the pollen development. The expression of *miR2118* was hardly detected during embryo sac development; however, 21 nt-phasiRNAs not only detected in pollen development but also in the embryo sac development, suggesting that the 21 nt-phasiRNAs was not the anthers specific phasiRNAs; probably synthesized in other tissues then transported to embryo sac. Additionally, the irregular distribution of pollen-specific-21 nt-phasiRNAs in autotetraploid rice was different from the results of Zhai et al. [26], who indicated that the 21 nt-phasiRNAs accumulated and peaked at pre-meiotic in maize. The expression patterns of 21 nt-phasiRNAs were more complicated in autotetraploid rice and remained unknown. Besides, the differentially expressed 24 nt-phasiRNAs were similar to the spatial-temporal expressions of *miR2275d* in 02428-4x pollen development. 24 nt-phasiRNAs were absent in the aberrant meiocytes of maize [26]. The down-regulated 24 nt-phasiRNAs and *miR2275d* were associated with the high frequency of cytological abnormalities in 02428-4x. We speculated that down-regulation of *miR2275* may result in the low expression of 24 nt-phasiRNAs, leading to the abnormal pollen mother cells development in autotetraploid rice.

### 24 nt TEs-siRNAs may cause genome instability and infertility in autotetraploid rice

The major function of 24 nt TEs-siRNAs was to maintain the genome integrity by silencing transposable elements. Many transposable elements may activate in response to “genomic shock” by polyploidization of rice. In autotetraploid rice, DNA methylation variation of transposable elements was found to play crucial role to adapt the whole-genome duplication (WGD) against the polyploidization [28]. Most of the down-regulated 24 nt siRNAs were associated with the accumulation of transcripts from DNA TEs in the *mop1* mutant, which may relate with the loss of function of RNA–Dependent RNA Polymerase 2 (RDR2) [61]. Here, we identified various kinds of 24 nt TEs-siRNAs associated with pollen and embryo sac development. In addition, the numbers of TEs-siRNAs were much higher during embryo sac development than pollen. The expression patterns of TEs-siRNAs were complex in different tissues of autotetraploid rice. Moreover, we found that ~50% TEs-siRNAs were differentially expressed in autotetraploid rice during pollen development and more than 80% of the differentially expressed TEs-siRNAs showed down-regulation, which indicated that reduction of 24 nt TEs-siRNAs in pollen development of autotetraploid rice may induce genome instability and infertility during the pollen development. These results were consistent with the allopolyploid wheat and *Arabidopsis*, where down-regulated TEs-siRNAs contribute to genome destabilization [62, 63]. However, 24 nt TEs-siRNAs were found to be up-regulated in embryo sac development. There was a great variation in the activity of RNA polymerase IV (Pol IV), DCL (Dicer-like) and Argonaute protein (AGO) in different development stage/tissue [64, 65], which resulted in differential expression patterns of TEs-siRNAs during pollen and embryo sac development. Moreover, siRNAs derived from transposable elements were similar to the nearby genes, which regulate the genes expression and play a crucial role in plant development [66, 67]. Many genes expression levels may change under the influence of TEs-siRNAs-triggered methylation, particularly at the crucial stages of female-meiosis, which explained more than 95% up-regulated TEs-siRNAs during the embryo sac development in autotetraploid rice.

## Conclusion

In the present study, we used high-throughput sequencing to identify the small RNA associated with the pollen and embryo sac development of autotetraploid and corresponding diploid rice. We identified 260 known and 488 novel miRNAs in autotetraploid and diploid rice, and 43 and 49 miRNAs specifically expressed during pollen and embryo sac development in autotetraploid rice, respectively. Various DEM were detected during pollen and embryo sac development in autotetraploid rice. Of these DEM, 18 and 21 important DEM were associated with male and female meiosis in autotetraploid rice, respectively. Moreover, abundantly expressed 21- and 24-nt phasiRNAs exhibited differential expressions during pollen development, and 21-nt phasiRNAs were also detected during embryo sac development in the present study. Interestingly, up-regulated 24-nt TEs-siRNAs were found to be dominant during the embryo sac development of autotetraploid rice, whereas a large proportion of down-regulated TEs-siRNAs were detected in pollen development. Consequently, the significant differential expression profiles of small RNAs in the autotetraploid rice may play important roles in regulating the development of pollen and embryo sac, thus resulting in low fertility and poor seed set in autotetraploid rice. Our findings provide a new insight on small RNA during pollen and embryo sac development in autotetraploid rice.

## Methods

### Rice material

Autotetraploid rice, 02428-4x, and its diploid rice (*Oryza sativa* L. subsp. *japonica*), 02428-2x, were used in this study. 02428-4x was developed from the chromosome doubling of 02428-2x by colchicine and was self-crossed for more than 25 generations in our lab. They were planted at the experimental farm of South China Agricultural University (SCAU) under field conditions. Anthers with four pollen development stages, including pre-meiotic interphase (PMA), meiosis (MA), single microspore stage (SCP) and bi-cellular pollen stage (BCP), were collected from 02428-4x and 02428-2x based on the floret length and DAPI fluorescence (Additional file 4: Table S23). The ovary tissues were collected with their corresponding embryo sac development stages (i.e. megasporocyte formation stage (MF), megasporocyte meiosis stage (MM), functional megaspore formation stage (FMF) and eight-nucleate embryo sac developing-stage (EES)). All the samples were stored at 4 °C for cytological observation and at −80 °C for RNA isolation.

### Analysis of agronomic/morphological traits

A total of eleven morphological traits, including number of effective panicles, thousand kernel weight, plant height, flag leaf length, flag leaf width, panicle length, grain length, grain width, seed set, pollen and embryo sac fertility were examined to determine the phenotypic variations in autotetraploid and diploid rice.

### Cytological observation

Anthers and ovaries were collected and fixed in Carnoy solution (ethanol: acetic acid = 3:1 v/v) for at least 24 h. The samples were stored in 70% ethanol at 4 °C after washing with 95% ethanol for two times. Chromosome behavior and configurations were identified and analyzed by acetocarmine squashing as described by Wu et al. [6]. Meiosis stages were classified and explained according to He et al. [7]. To investigate the variation in pollen development of 02428-2x and 02428-4x, a whole mount eosin B confocal laser scanning microscopy (WE-CLSM) was used. The samples were observed according to Wu et al. [6] with some minor modifications. The dissected anthers were hydrated in 50%, 30% ethanol and distilled water for 5 min each. After an eosin B staining procedure for 30 min, the samples were dehydrated in a series of ethanol solutions (10%, 30%, 50%, 70%, 90% and 100% ethanol) for 5 min. Finally, the dehydrated samples were transferred into a mixture of ethanol and methyl salicylate (1:1) for 1 h, and then stored in pure methyl salicylate and observed under WE-CLSM. To investigate the variations in embryo sac development of 02428-2x and 02428-4x, WE-CLSM was used again and followed the procedure as described by Zeng et al. [68].

### Small RNA library construction and sequencing

Total RNA was extracted from the anthers and ovaries by using Trizol reagent (Invitrogen, Carlsbad, CA, USA). Illumina’s TruSeq small RNA sample preparation Kits (San Diego, CA, USA) were used to construct small RNA libraries. Then the small sequencing (36 bp) was performed on an Illumina Hiseq2500 at the LC-BIO (Hangzhou, China).

### Data processing/analysis of miRNAs

After sequencing, the raw data was further processed to remove common RNA families (rRNA, tRNA, snRNA, snoRNA), repeats, low complexity, adapter dimers and junk by ACGT101-miR [10]. Then, the unique sequences (18–25 nt in length) were BLASTed to miRBase 20.0 (ftp://mirbase.org/pub/mirbase/) to identify known miRNAs. The novel miRNAs were predicted by using RNAfold software (http://rna.tbi.univie.ac.at/cgi-bin/RNAWebSuite/RNAfold.cgi). The miRNAs were considered as confidence miRNAs according to the normalized deep-sequencing levels (with the exclusion of 10 RPM) in all anthers and ovaries libraries. miRNAs with *P*-value (Fisher exact test and Chi-square (*X*2) test) < 0.05 and |log2 (fold change ratio)| > 1 were considered as differentially expressed miRNAs. Targeted genes of differentially expressed miRNAs were predicted by using TargetFinder [69]. GO enrichment analysis and Protein-protein interaction networks were performed using AgriGO (http://bioinfo.cau.edu.cn/agriGO/) [70] and STRING (http://string-db.org/) [71], respectively.

### Data processing/analysis of phasiRNAs

We used PhaseTank to systemically characterize the 21 and 24 nt-phasiRNAs [41]. Then the confidence phasiRNAs with phasing scores higher than or equal to 25 were selected [26], and filtered the phasiRNAs that were triggered by *miR2118* and *miR2275* families. Finally, it yielded 1117 loci generating 21 nt-phasiRNAs and 190 loci generating 24 nt-phasiRNAs. PhasiRNAs with *P*-value (Fisher exact test) < 0.05 and |log2 (fold change ratio)| > 1 were considered as differentially expressed phasiRNAs.

### Data processing/analysis of TEs-siRNAs

To find out the siRNAs associated with the transposable elements, we followed the method described by Zhang et al. [28]. Firstly, we filtered the reads that did not match miRNA, rRNA, tRNA, snRNA, or snoRNA, and then selected the reads which mapped to the transposable elements of rice reference genome. These subsequent of siRNAs were considered as TEs-siRNAs, and used for further analysis. The classifications of TEs-siRNAs were done by Li et al. [10]. The differentially expressed TEs-siRNAs were identified by the conditions of *P*-value (Fisher exact test) < 0.05 and |log2 (fold change ratio)| > 1.

### Quantitative real-time PCR (qRT-PCR) analysis

The total RNA from anthers and ovaries were used as template for reverse transcription with miRNA-specific stem-loop RT primers [72] by the Transcriptor First Strand cDNA Synthesis Kit (Roche). The reaction was as follow: incubated for 30 min at 16 °C, followed by pulsed RT of 60 cycles at 30 °C for 30 s, 42 °C for 30 s and 50 °C for 1 s, and a final incubation at 85 °C for 5 min to inactivate the reverse transcriptase [73]. The qRT-PCRs were performed on Lightcycler480 (Roche) using the SsoAdvanced Universal SYBR Green Supermix (Bio-RAD). The reaction conditions were as follows: 30 s at 95 °C, 40 cycles of 95 °C denaturation for 5 s and 58 °C annealing and extension for 20 s. Three biological replications were performed in this experiment. *U6* snRNA was used as an internal reference for qRT-PCR of miRNAs. The relative expression of miRNAs was calculated by the *2* ^*–ΔΔCT*^ method [74]. The stem-loop RT primers were designed by the Primer premier 5.0 software (Additional file 4: Table S24).

## Additional files

Additional file 1: Figure S1. Chromosome observations of the diploid and autotetraploid rice. **Figure S2.** Cytological observation of pollen development in 02428-4x. **Figure S3.** Chromosome behavior during pollen mother cell (PMC) meiosis in 02428-4x. **Figure S4.** Cytological observation of embryo sac development in 02428-4x. **Figure S5.** Principal component analysis in each library. **Figure S6.** Validation of the miRNAs in 02428-4x and 02428-2x of pollen and embryo sac at each development stage. **Figure S7.** Venn analysis of the DEM (differentially expressed miRNAs) between pollen and embryo sac development in autotetraploid rice. **Figure S8.** Classification of the differentially expressed miRNAs between embryo sac and pollen development in autotetraploid rice. **Figure S9.** GO (Gene Ontology) enrichment analysis of predicted targets of DEM-P (pollen-enriched miRNAs) in autotetraploid rice. **Figure S10.** GO enrichment analysis of predicted targets of DEM-ES (embryo sac-enriched miRNAs) in autotetraploid rice. **Figure S11.** GO enrichment analysis of predicted targets of differentially expressed miRNAs in MA (meiosis). **Figure S12.** GO enrichment analysis of predicted targets of differentially expressed miRNAs in MM (megasporocyte meiosis stage). **Figure S13.** Protein interactions between the targets of DEM (differentially expressed miRNAs) and meiosis-related genes. **Figure S14.** Distribution of TEs-siRNAs (siRNAs associated with transposable elements) in pollen and embryo sac development. (PDF 2706 kb)
Additional file 2: Table S1. Main agronomic traits of autotetraploid (02428-4x) and diploid (02428-2x) rice. **Table S2.** Frequencies of pollen fertility and embryo sac fertility in 02428-4x and 02428-2x. **Table S3.** The development of pollen and embryo sac in 02428-2x and 02428-4x. **Table S4.** Frequency of abnormalities in autotetraploid and diploid rice during the pollen development. **Table S5.** Observations of chromosome behavior and configurations between autotetraploid rice and diploid rice. **Table S6.** Summary of the known and predicted miRNAs in 02428-4x and 02428-2x. **Table S7.** miRNAs detected during pollen and embryo sac development of 02428-4x and 02428-2x. **Table S8.** The specific miRNAs found in each library (Development stages). (XLSX 214 kb)
Additional file 3: Table S9. Differentially expressed miRNAs (DEM) between diploid and autotetraploid during pollen development. **Table S10.** Differentially expressed miRNAs (DEM) between diploid and autotetraploid rice during embryo sac development. **Table S11.** Comparison of the differentially expressed miRNAs (DEM) between the pollen and embryo sac development in autotetraploid rice. **Table S12.** Differentially expressed miRNAs between embryo sac and pollen development in autotetraploid rice. **Table S13.** DEM-P (pollen-enriched miRNAs) and DEM-ES (embryo sac-enriched miRNAs) in 02428-4x. **Table S14.** Overall information about the predicted targets of detected miRNAs by Targetfinder. **Table S15.** Comparison of targets predicted by differentially expressed miRNAs with the previous studies on MA (meiosis). **Table S16.** Comparison of targets predicted by differentially expressed miRNAs with the previous studies on MM (megasporocyte meiosis stage). (XLSX 417 kb)
Additional file 4: Table S17. Protein-protein interaction of meiosis-related genes with the targets predicted by the DEM associated with meiosis. **Table S18.** 21 nt-phasiRNAs triggered by the *miR2118*. **Table S19.** 24 nt-phasiRNAs triggered by the *miR2275*. **Table S20.** Overview of 24 nt TEs-siRNAs during pollen and embryo sac development of 02428-4x and 02428-2x. **Table S21.** Distribution of 24 nt TEs-siRNAs in autotetraploid and diploid rice. **Table S22.** Differentially expressed 24 nt TEs-siRNAs during pollen and embryo sac development of autotetraploid rice. **Table S23.** Anther length during pollen development stages in autotetraploid and diploid rice. **Table S24.** The stem–loop RT primers used in the present study. (XLSX 1012 kb)

## Abbreviations

BCP: (bi-cellular pollen stage)
DEM: (differentially expressed miRNAs)
EES: (eight-nucleate embryo sac developing-stage)
EMC: Embryo sac mother cell
FMF: Functional megaspore formation stage
MA: Meiosis
MF: Megasporocyte formation stage
MM: Megasporocyte meiosis stage
PMA: Pre-meiotic interphase
PMC: Pollen mother cell
RPM: Reads per million
SCP: Single microspore stage

## References

[1] Shahid MQ, Xu H, Lin S, Chen Z, Naeem M, Li Y (2012). Genetic analysis and hybrid vigor study of grain yield and other quantitative traits in autotetraploid rice. Pak. J. Bot..

[2] Wu J, Hu C, Shahid MQ, Guo H, Zeng Y, Liu X (2013). Analysis on genetic diversification and heterosis in autotetraploid rice. Springerplus.

[3] Shahid MQ, Li Y, Saleem MF, Naeem M, Wei C, Liu X (2013). Yield and yield components in autotetraploid and diploid rice genotypes (*indica* and *japonica*) sown in early and late seasons. Aust. J. Crop Sci..

[4] Guo H, Lu Y, Feng J, Yang B, Liu X (2006). Further observation on the formation and development of autotetraploid rice embryo sac using laser scanning confocal microscopy. Acta Laser Biology Sinica.

[5] Shahid MQ, Sun J, Wei C, Zhang P, Liu X (2010). Studies on the abnormality of embryo sac and pollen fertility in autotetraploid rice during different growing seasons. Pak. J. Bot..

[6] Wu J, Shahid MQ, Guo H, Yin W, Chen Z, Wang L (2014). Comparative cytological and transcriptomic analysis of pollen development in autotetraploid and diploid rice. Plant Reprod..

[7] He J, Shahid MQ, Chen Z, Chen X, Liu X, Lu Y (2011). Abnormal PMC microtubule distribution pattern and chromosome behavior resulted in low pollen fertility of an intersubspecific autotetraploid rice hybrid. Plant Syst. Evol..

[8] Kubo T, Fujita M, Takahashi H, Nakazono M, Tsutsumi N, Kurata N (2013). Transcriptome analysis of developing ovules in rice isolated by laser microdissection. Plant Cell Physiol..

[9] Wu Y, Yang L, Cao A, Wang J (2015). Gene expression profiles in rice developing ovules provided evidence for the role of sporophytic tissue in female gametophyte development. PLoS One.

[10] Li X, Shahid M, Wu J, Wang L, Liu X, Lu Y (2016). Comparative small RNA analysis of pollen development in autotetraploid and diploid rice. Int. J. Mol. Sci..

[11] Boke H, Ozhuner E, Turktas M, Parmaksiz I, Ozcan S, Unver T (2015). Regulation of the alkaloid biosynthesis by miRNA in opium poppy. Plant Biotechnol. J..

[12] Hong Y, Jackson S (2015). Floral induction and flower formation-the role and potential applications of miRNAs. Plant Biotechnol. J..

[13] Shriram V, Kumar V, Devarumath RM, Khare TS, Wani SH (2016). MicroRNAs as potential targets for abiotic stress tolerance in plants. Front. Plant Sci..

[14] Xie F, Jones DC, Wang Q, Sun R, Zhang B (2015). Small RNA sequencing identifies miRNA roles in ovule and fibre development. Plant Biotechnol. J..

[15] Wei L, Yan L, Wang T (2011). Deep sequencing on genome-wide scale reveals the unique composition and expression patterns of microRNAs in developing pollen of *Oryza sativa*. Genome Biol..

[16] Peng H, Chun J, Ai T, Tong Y, Zhang R, Zhao M (2012). MicroRNA profiles and their control of male gametophyte development in rice. Plant Mol. Biol..

[17] Yan J, Zhang H, Zheng Y, Ding Y (2015). Comparative expression profiling of miRNAs between the cytoplasmic male sterile line MeixiangA and its maintainer line MeixiangB during rice anther development. Planta.

[18] Peng T, Sun H, Du Y, Zhang J, Li J, Liu Y (2013). Characterization and expression patterns of microRNAs involved in rice grain filling. PLoS One.

[19] Yi R, Zhu Z, Hu J, Qian Q, Dai J, Ding Y (2013). Identification and expression analysis of microRNAs at the grain filling stage in rice (*Oryza sativa L.*) via deep sequencing. PLoS One.

[20] Millar AA, Gubler F (2005). The *Arabidopsis* GAMYB-like genes, *MYB33* and *MYB65*, are microRNA-regulated genes that redundantly facilitate anther development. Plant Cell.

[21] Palatnik JF, Wollmann H, Schommer C, Schwab R, Boisbouvier J, Rodriguez R (2007). Sequence and expression differences underlie functional specialization of *Arabidopsis* microRNAs *miR159* and *miR319*. Dev. Cell.

[22] Zhu Q, Upadhyaya NM, Gubler F, Helliwell CA (2009). Over-expression of *miR172* causes loss of spikelet determinacy and floral organ abnormalities in rice (*Oryza sativa*). BMC Plant Biol..

[23] Zhang Y, Yu Y, Wang C, Li Z, Liu Q, Xu J (2013). Overexpression of microRNA *OsmiR397* improves rice yield by increasing grain size and promoting panicle branching. Nat. Biotechnol..

[24] Wu MF, Tian Q, Reed JW (2006). *Arabidopsis* microRNA167 controls patterns of *ARF6* and *ARF8* expression, and regulates both female and male reproduction. Development.

[25] Komiya R, Ohyanagi H, Niihama M, Watanabe T, Nakano M, Kurata N (2014). Rice germline-specific Argonaute MEL1 protein binds to phasiRNAs generated from more than 700 lincRNAs. Plant J..

[26] Zhai J, Zhang H, Arikit S, Huang K, Nan G, Walbot V (2015). Spatiotemporally dynamic, cell-type-dependent premeiotic and meiotic phasiRNAs in maize anthers. Proc. Natl. Acad. Sci. USA.

[27] Chen C, Farmer AD, Langley RJ, Mudge J, Crow JA, May GD (2010). Meiosis-specific gene discovery in plants: RNA-Seq applied to isolated *Arabidopsis* male meiocytes. BMC Plant Biol..

[28] Zhang J, Liu Y, Xia E, Yao Q, Liu X, Gao L (2015). Autotetraploid rice methylome analysis reveals methylation variation of transposable elements and their effects on gene expression. Proc. Natl. Acad. Sci. USA.

[29] Fujita M, Horiuchi Y, Ueda Y, Mizuta Y, Kubo T, Yano K (2010). Rice expression atlas in reproductive development. Plant Cell Physiol..

[30] Deveshwar P, Bovill WD, Sharma R, Able JA, Kapoor S (2011). Analysis of anther transcriptomes to identify genes contributing to meiosis and male gametophyte development in rice. BMC Plant Biol..

[31] Jin Y, Yang H, Wei Z, Ma H, Ge X (2013). Rice male development under drought stress: phenotypic changes and stage-dependent transcriptomic reprogramming. Mol. Plant.

[32] Aya K, Ueguchi-Tanaka M, Kondo M, Hamada K, Yano K, Nishimura M (2009). Gibberellin modulates anther development in rice via the transcriptional regulation of GAMYB. Plant Cell.

[33] Wu J, Shahid MQ, Chen L, Chen Z, Wang L, Liu X (2015). Polyploidy enhances F_1_ pollen sterility loci interactions that increase meiosis abnormalities and pollen sterility in autotetraploid rice. Plant Physiol..

[34] Wei LQ, Xu WY, Deng ZY, Su Z, Xue Y, Wang T (2010). Genome-scale analysis and comparison of gene expression profiles in developing and germinated pollen in *Oryza sativa*. BMC Genomics.

[35] Honys D, Twell D (2004). Transcriptome analysis of haploid male gametophyte development in *Arabidopsis*. Genome Biol..

[36] Yant L, Hollister JD, Wright KM, Arnold BJ, Higgins JD, Franklin FC (2013). Meiotic adaptation to genome duplication in *Arabidopsis arenosa*. Curr. Biol..

[37] Wright KM, Arnold B, Xue K, Urinova M, O'Connell J, Bomblies K (2015). Selection on meiosis genes in diploid and tetraploid *Arabidopsis arenosa*. Mol. Biol. Evol..

[38] Ohnishi T, Takanashi H, Mogi M, Takahashi H, Kikuchi S, Yano K (2011). Distinct gene expression profiles in egg and synergid cells of rice as revealed by cell type-specific microarrays. Plant Physiol..

[39] Schmidt A, Wuest SE, Vijverberg K, Baroux C, Kleen D, Grossniklaus U (2011). Transcriptome analysis of the *Arabidopsis* megaspore mother cell uncovers the importance of RNA helicases for plant germline development. PLoS Biol..

[40] Wang C, Wang Y, Cheng Z, Zhao Z, Chen J, Sheng P (2016). The role of *OsMSH4* in male and female gamete development in rice meiosis. J. Exp. Bot..

[41] Guo Q, Qu X, Jin W (2015). PhaseTank: genome-wide computational identification of phasiRNAs and their regulatory cascades. Bioinformatics.

[42] Fan G, Zhai X, Niu S, Ren Y (2014). Dynamic expression of novel and conserved microRNAs and their targets in diploid and tetraploid of *Paulownia tomentosa*. Biochimie.

[43] Niu S, Fan G, Xu E, Deng M, Zhao Z, Dong Y (2014). Transcriptome/Degradome-wide discovery of microRNAs and transcript targets in two *Paulownia australis* genotypes. PLoS One.

[44] Niu S, Fan G, Zhao Z, Deng M, Dong Y (2014). High-throughput sequencing and degradome analysis reveal microRNA differential expression profiles and their targets in *Paulownia fortunei*. Plant Cell Tiss. Org..

[45] Zhai X, Niu S, Ren Y, Fan G (2016). Discovery and profiling of microRNAs and their targets in *Paulownia* ‘Yuza 1’ plants via high-throughput sequencing and degradome analysis. Genes Genom..

[46] Chagué V, Just J, Mestiri I, Balzergue S, Tanguy AM, Huneau C (2010). Genome-wide gene expression changes in genetically stable synthetic and natural wheat allohexaploids. New Phytol..

[47] Chelaifa H, Chagué V, Chalabi S, Mestiri I, Arnaud D, Deffains D (2013). Prevalence of gene expression additivity in genetically stable wheat allohexaploids. New Phytol..

[48] Yoo MJ, Szadkowski E, Wendel JF (2013). Homoeolog expression bias and expression level dominance in allopolyploid cotton. Heredity.

[49] Li X (2016). Natural attributes and agricultural implications of somatic genome variation. Curr. Issues Mol. Biol..

[50] Nazzicari N, Biscarini F, Cozzi P, Brummer EC, Annicchiarico P (2016). Marker imputation efficiency for genotyping-by-sequencing data in rice (*Oryza sativa*) and alfalfa (*Medicago sativa*). Mol. Breeding.

[51] Ha M, Lu J, Tian L, Ramachandran V, Kasschau KD, Chapman EJ (2009). Small RNAs serve as a genetic buffer against genomic shock in *Arabidopsis* interspecific hybrids and allopolyploids. Proc. Natl. Acad. Sci. USA.

[52] Xie F, Zhang B (2015). microRNA evolution and expression analysis in polyploidized cotton genome. Plant Biotechnol. J.

[53] Zhang H, Zhao H, Wu S, Huang F, Wu K, Zeng X (2016). Global methylation patterns and their relationship with gene expression and small RNA in rice lines with different ploidy. Front. Plant Sci..

[54] Tsuji H, Aya K, Ueguchi-Tanaka M, Shimada Y, Nakazono M, Watanabe R (2006). *GAMYB* controls different sets of genes and is differentially regulated by microRNA in aleurone cells and anthers. Plant J..

[55] Quilichini TD, Grienenberger E, Douglas CJ (2015). The biosynthesis, composition and assembly of the outer pollen wall: A tough case to crack. Phytochemistry.

[56] Wang Y, Lin YC, So J, Du Y, Lo C (2013). Conserved metabolic steps for sporopollenin precursor formation in tobacco and rice. Physiol. Plant.

[57] Aklilu BB, Soderquist RS, Culligan KM (2014). Genetic analysis of the Replication Protein A large subunit family in *Arabidopsis* reveals unique and overlapping roles in DNA repair, meiosis and DNA replication. Nucleic Acids Res..

[58] Johnson C, Kasprzewska A, Tennessen K, Fernandes J, Nan GL, Walbot V (2009). Clusters and superclusters of phased small RNAs in the developing inflorescence of rice. Genome Res..

[59] Zhai J, Jeong DH, De Paoli E, Park S, Rosen BD, Li Y (2011). MicroRNAs as master regulators of the plant NB-LRR defense gene family via the production of phased, trans-acting siRNAs. Genes Dev..

[60] Song X, Li P, Zhai J, Zhou M, Ma L, Liu B (2012). Roles of *DCL4* and *DCL3b* in rice phased small RNA biogenesis. Plant J..

[61] Jia Y, Lisch DR, Ohtsu K, Scanlon MJ, Nettleton D, Schnable PS (2009). Loss of RNA-dependent RNA polymerase 2 (RDR2) function causes widespread and unexpected changes in the expression of transposons, genes, and 24-nt small RNAs. PLoS Genet..

[62] Kenan-Eichler M, Leshkowitz D, Tal L, Noor E, Melamed-Bessudo C, Feldman M (2011). Wheat hybridization and polyploidization results in deregulation of small RNAs. Genetics.

[63] Slotkin RK, Vaughn M, Borges F, Tanurdzic M, Becker JD, Feijo JA (2009). Epigenetic reprogramming and small RNA silencing of transposable elements in pollen. Cell.

[64] Mosher RA, Melnyk CW, Kelly KA, Dunn RM, Studholme DJ, Baulcombe DC (2009). Uniparental expression of PolIV-dependent siRNAs in developing endosperm of *Arabidopsis*. Nature.

[65] Wierzbicki AT, Haag JR, Pikaard CS (2008). Noncoding transcription by RNA polymerase Pol IVb/Pol V mediates transcriptional silencing of overlapping and adjacent genes. Cell.

[66] Liu J, He Y, Amasino R, Chen X (2004). siRNAs targeting an intronic transposon in the regulation of natural flowering behavior in *Arabidopsis*. Genes Dev.

[67] Henderson IR, Jacobsen SE (2008). Tandem repeats upstream of the *Arabidopsis* endogene SDC recruit non-CG DNA methylation and initiate siRNA spreading. Genes Dev..

[68] Zeng Y, Hu C, Lu Y, Li J, Liu X (2007). Diversity of abnormal embryo sacs in *indica/japonica* hybrids in rice demonstrated by confocal microscopy of ovaries. Plant Breeding.

[69] Fahlgren N, Carrington JC (2010). miRNA target prediction in plants. Methods Mol. Biol.

[70] Du Z, Zhou X, Ling Y, Zhang Z, Su Z (2010). agriGO: a GO analysis toolkit for the agricultural community. Nucleic Acids Res.

[71] Szklarczyk D, Franceschini A, Wyder S, Forslund K, Heller D, Huerta-Cepas J (2015). STRING v10: protein-protein interaction networks, integrated over the tree of life. Nucleic Acids Res..

[72] Chen C, Ridzon DA, Broomer AJ, Zhou Z, Lee DH, Nguyen JT (2005). Real-time quantification of microRNAs by stem-loop RT-PCR. Nucleic Acids Res..

[73] Varkonyi-Gasic E, Wu R, Wood M, Walton EF, Hellens RP (2007). Protocol: a highly sensitive RT-PCR method for detection and quantification of microRNAs. Plant Methods.

[74] Livak KJ, Schmittgen TD (2001). Analysis of relative gene expression data using real-time quantitative PCR and the 2^*–ΔΔCT*^ Method. Methods.

