# Supplementary material for: Analysis of small RNAs revealed differential expressions during pollen and embryo sac development in autotetraploid rice
Source: BMC Genomics. 2017 Feb 6;18:129. doi: 10.1186/s12864-017-3526-8 (PMC5295217; doi:10.1186/s12864-017-3526-8)
Supplement: Additional file 1: Figure S1. — Chromosome observations of the diploid and autotetraploid rice. Figure S2. Cytological observation of pollen development in 02428-4x. Figure S3. Chromosome behavior during pollen mother cell (PMC) meiosis in 02428-4x. Figure S4. Cytological observation of embryo sac development in 02428-4x. Figure S5. Principal component analysis in each library. Figure S6. Validation of the miRNAs in 02428-4x and 02428-2x of pollen and embryo sac at each development stage. Figure S7. Venn analysis of the DEM (differentially expressed miRNAs) between pollen and embryo sac development in autotetraploid rice. Figure S8. Classification of the differentially expressed miRNAs between embryo sac and pollen development in autotetraploid rice. Figure S9. GO (Gene Ontology) enrichment analysis of predicted targets of DEM-P (pollen-enriched miRNAs) in autotetraploid rice. Figure S10. GO enrichment analysis of predicted targets of DEM-ES (embryo sac-enriched miRNAs) in autotetraploid rice. Figure S11. GO enrichment analysis of predicted targets of differentially expressed miRNAs in MA (meiosis). Figure S12. GO enrichment analysis of predicted targets of differentially expressed miRNAs in MM (megasporocyte meiosis stage). Figure S13. Protein interactions between the targets of DEM (differentially expressed miRNAs) and meiosis-related genes. Figure S14. Distribution of TEs-siRNAs (siRNAs associated with transposable elements) in pollen and embryo sac development. (PDF 2706 kb) [file 12864_2017_3526_MOESM1_ESM.pdf]

Additional file 1

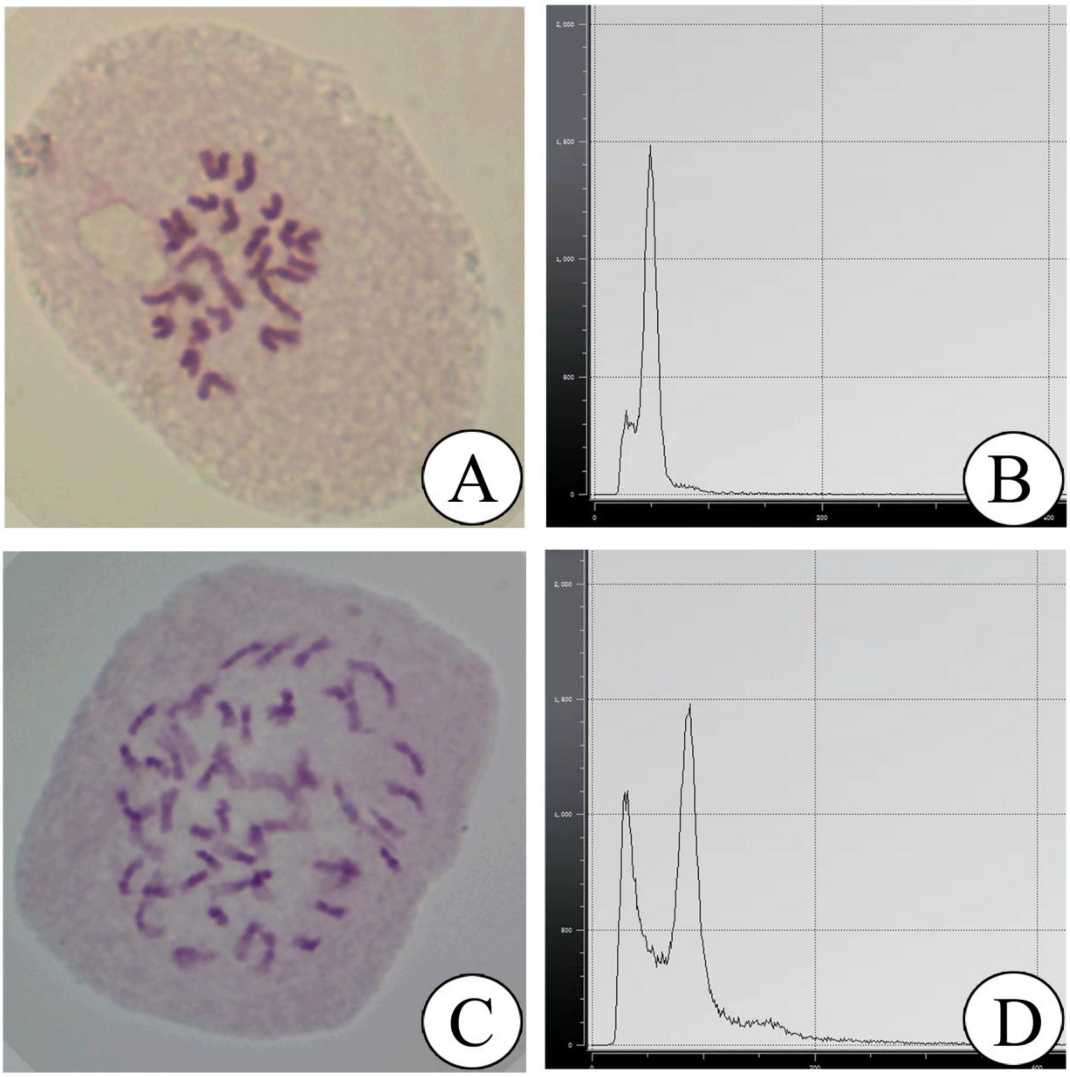

Fig. S1 Chromosome observations of the diploid and autotetraploid rice. (A, C) Chromosomes of 02428-2x and 02428-4x. (B, D) Flow cytometry analysis showed that DNA content of 02428-4x is two-fold higher than 02428-2x.

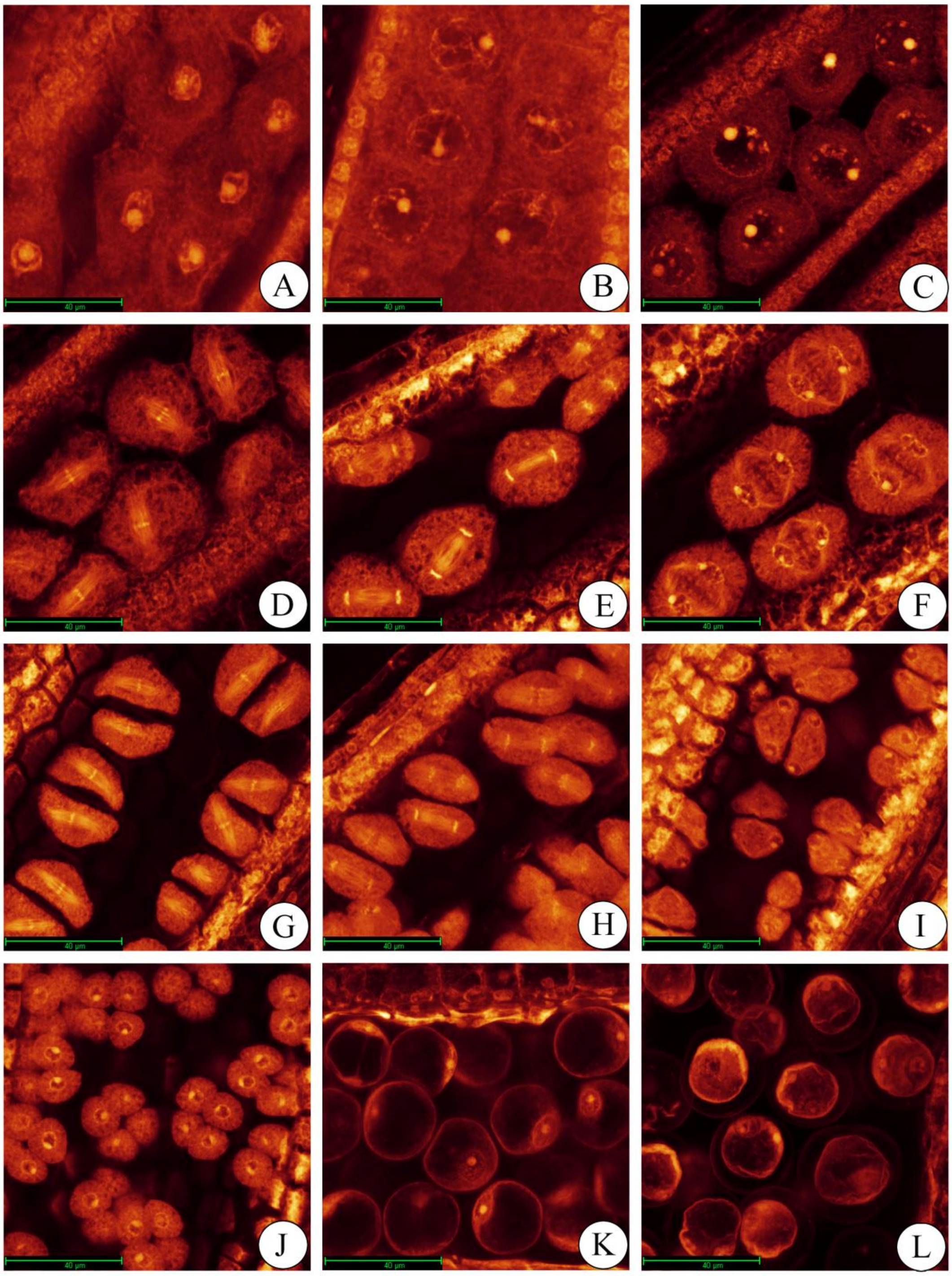

Fig. S2 Cytological observation of pollen development in 02428-4x. (A) pollen mother cell formation. (B-J) pollen mother cell meiosis stages (pachytene (B), diakinesis (C), metaphase I (D), anaphase I (E), prophase II (F), metaphase II (G), anaphase II (H), telophase II (I) and tetrad stage (J)). (K) single microspore stage. (L) bi-cellular pollen stage. Bars=40 μm.

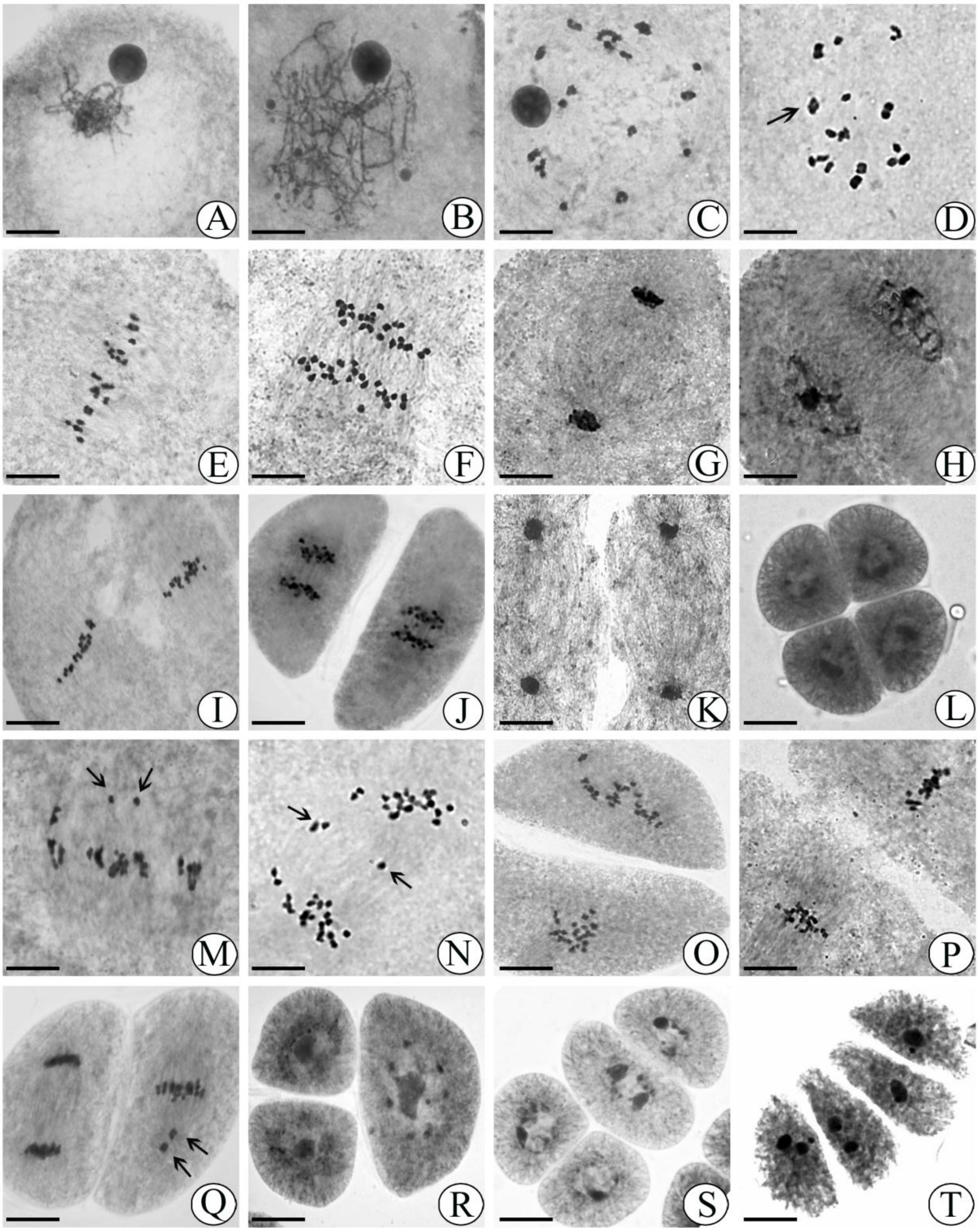

Fig. S3 Chromosome behavior during pollen mother cell (PMC) meiosis in 02428-4x. (A) zygotene. (B) pachytene. (C) diplotene. (D) diakinesis. Arrows are indicating multivalent. (E) metaphase I. (F) anaphase I. (G) telophase I. (H) prophase II. (I) metaphase II. (J) anaphase II. (K) telophase II. (L) tetrad stage. (M) abnormal metaphase I, showing straggled chromosomes (arrow). (N) abnormal anaphase I, showing lagged chromosomes (arrow). (O, P) abnormal spindle in metaphase II. (Q) abnormal PMC in dyad, showing asynchrony of the dyad, with one at metaphase II and another at anaphase II, arrows indicate the straggling quadrivalents. (R) triad. (S) 'T' type of tetrad. (T) linear type of tetrad. Bars=10 μm.

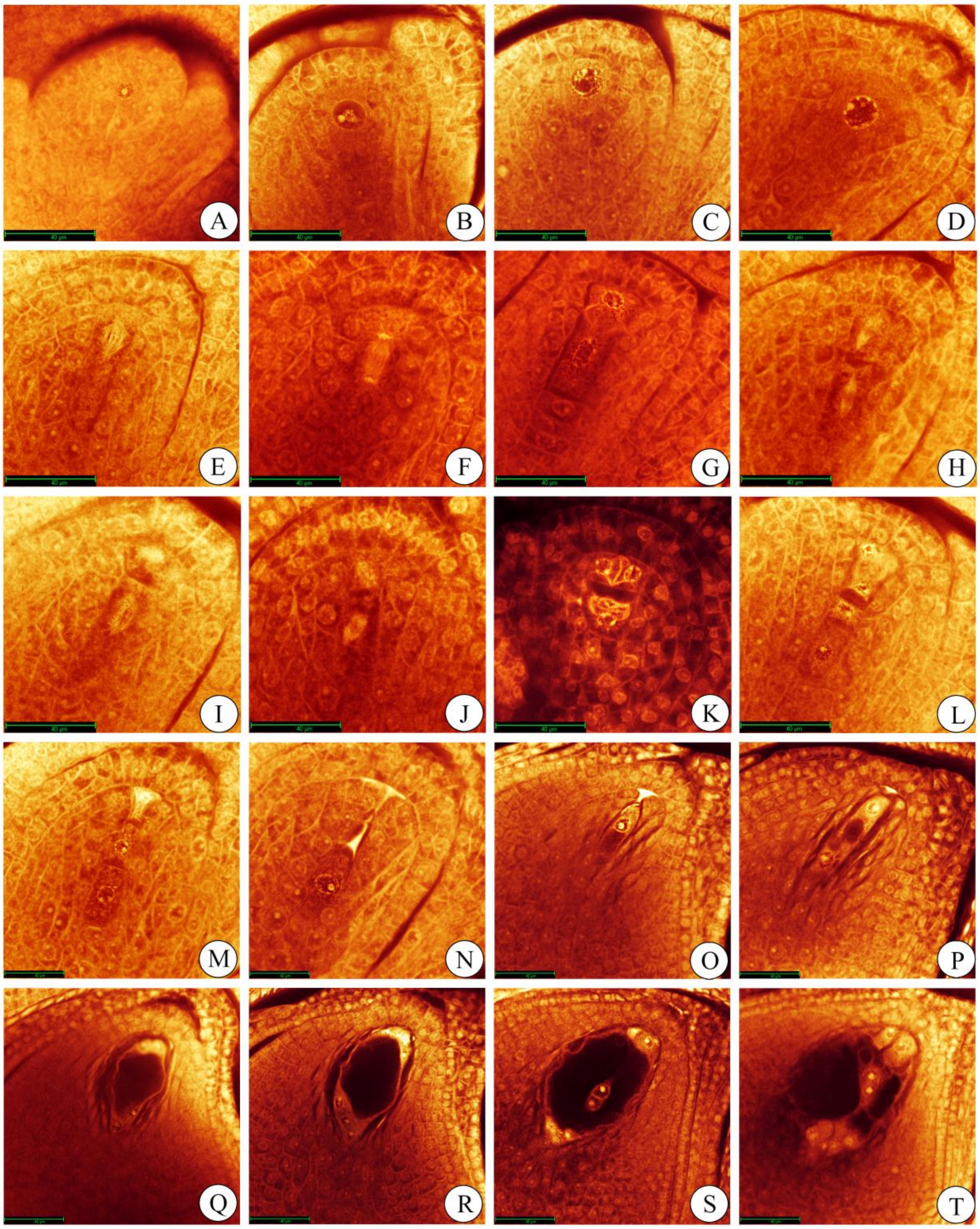

Fig. S4 Cytological observation of embryo sac development in 02428-4x. (A) megasporocyte formation stage. (B-L) megasporocyte meiosis stage, different types of tetrads (I-K). (M, N) functional megaspore formation stage. (O) mono-nucleate embryo sac formation stage. (P, Q) embryo sac mitosis stage. (R-T) eight-nucleate embryo sac developing-stage. Bars=40 μm.

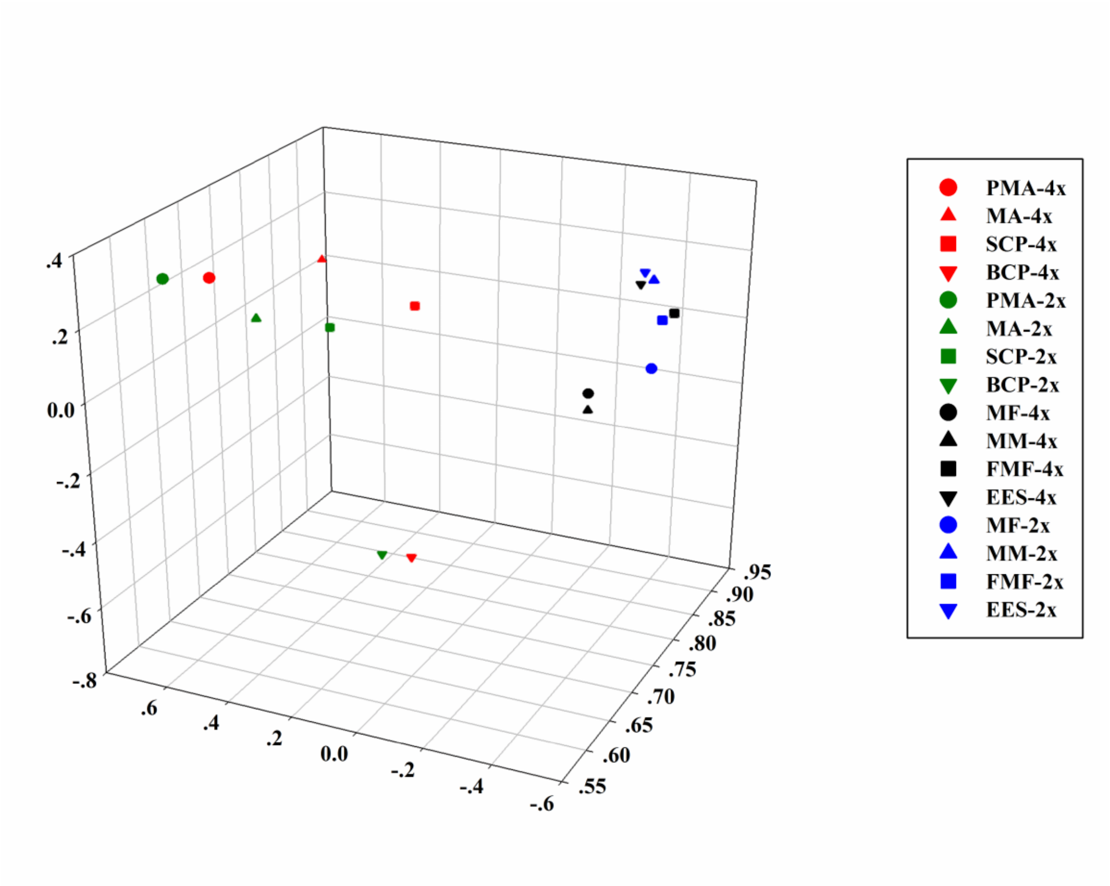

Fig. S5 Principal component analysis in each library. PMA, MA, SCP and BCP represent pre-meiotic interphase, meiosis and single microspore stage and bi-cellular pollen stage, respectively. MF, MM, FMF and EES represent megasporocyte formation stage, megasporocyte meiosis stage, functional megaspore formation stage and eight-nucleate embryo sac developing-stage. “4x” and “2x” represent the autotetraploid and diploid rice.

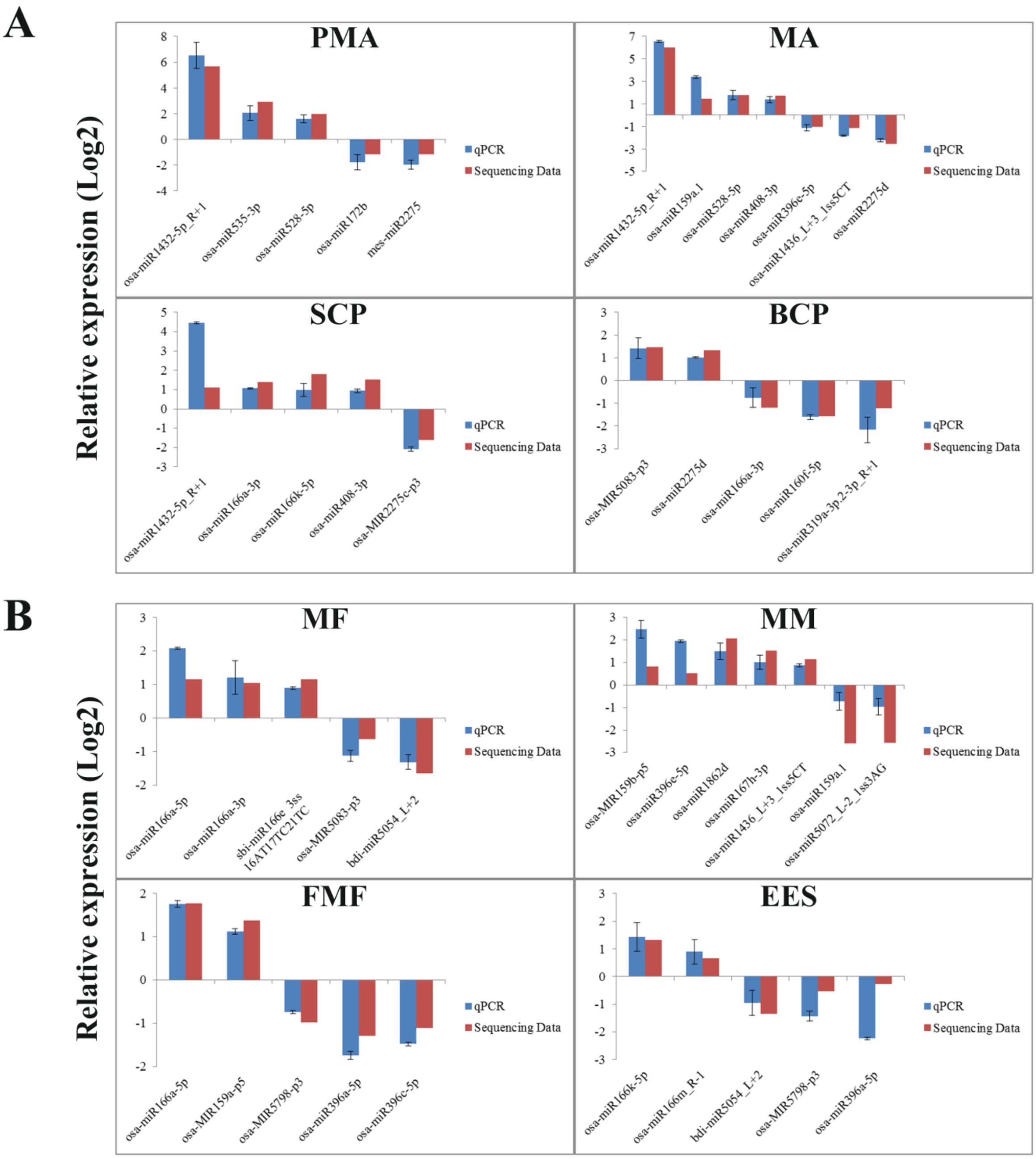

Fig. S6 Validation of the miRNAs in 02428-4x and 02428-2x of pollen and embryo sac at each development stage. PMA, MA, SCP and BCP represent pre-meiotic interphase, meiosis and single microspore stage and bi-cellular pollen stage, respectively. MF, MM, FMF and EES represent megasporocyte formation stage, megasporocyte meiosis stage, functional megaspore formation stage and eight-nucleate embryo sac developing-stage. “4x” and “2x” represent the autotetraploid and diploid rice. *U6* snRNA was used as an internal reference for the qRT-PCR. The X- and Y-axis are repressing the miRNAs and relative expression levels, respectively. Error bars indicate the standard deviation (SD) of three biological replicates.

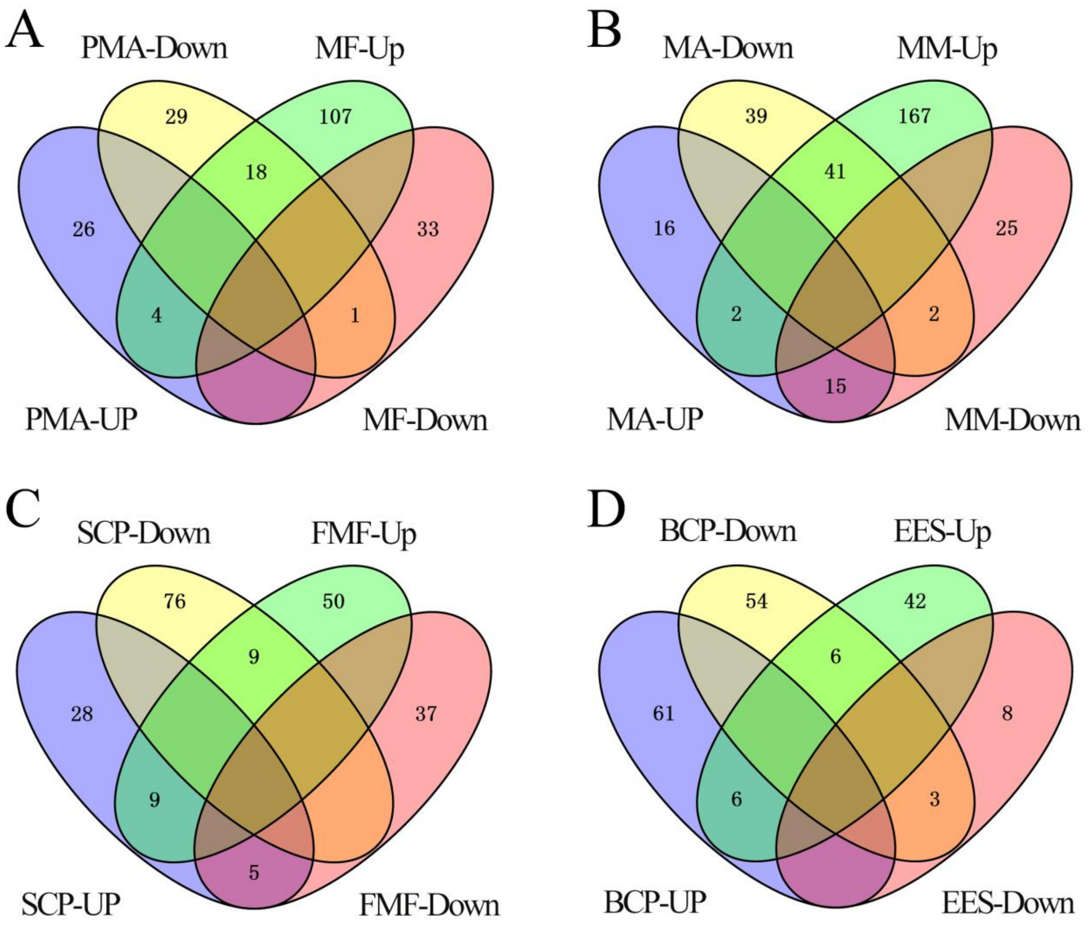

Fig. S7 Venn analysis of the DEM (differentially expressed miRNAs) between pollen and embryo sac development in autotetraploid rice. PMA, MA, SCP and BCP represent pre-meiotic interphase, meiosis and single microspore stage and bi-cellular pollen stage, respectively. MF, MM, FMF and EES represent megasporocyte formation stage, megasporocyte meiosis stage, functional megaspore formation stage and eight-nucleate embryo sac developing-stage. Up and Down represent the up-regulated and down-regulated miRNAs in autotetraploid rice.

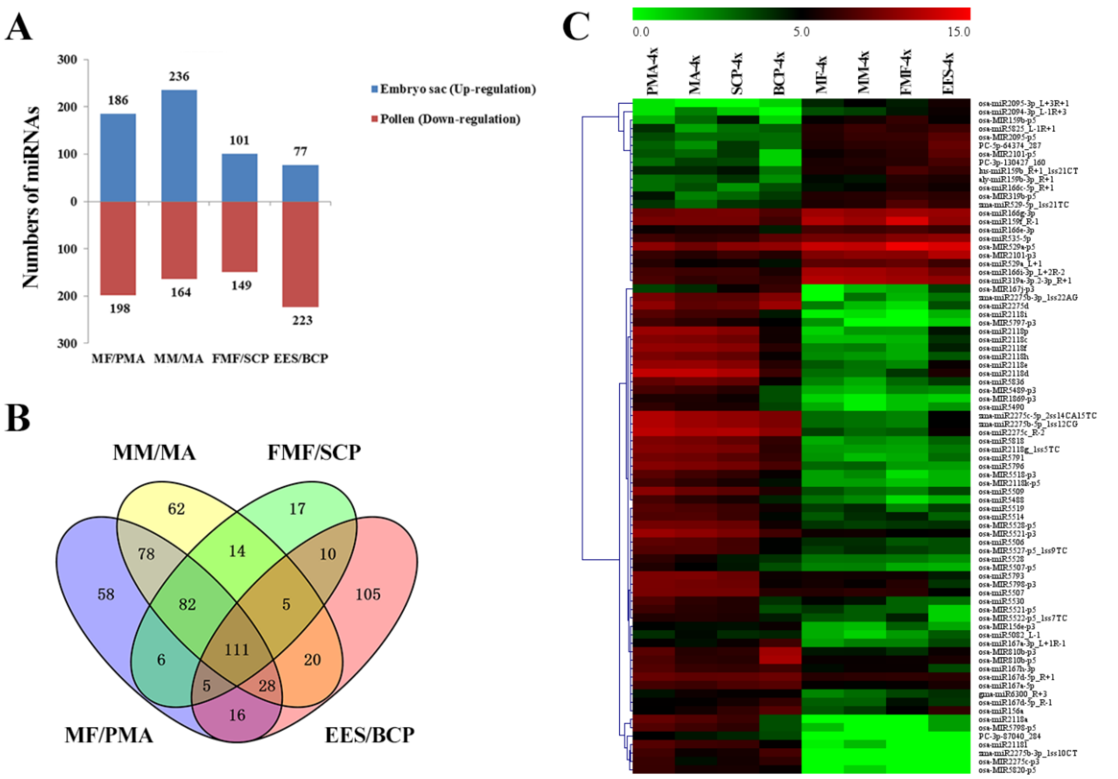

Fig. S8 Classification of the differentially expressed miRNAs between embryo sac and pollen development in autotetraploid rice. (A) The number of DEM at each development stage. (B) Venn analysis of the DEM at each stage of autotetraploid. (C) Hierarchical cluster analysis of the anther-enriched miRNAs and ovary-enriched miRNAs in 02428-4x. The hierarchical clustering tree in different libraries of pollen and embryo sac development was constructed by MultiExperiment View (version 4.9). Red represented the high expression levels of miRNAs. We could clearly distinguish the anther-enriched miRNAs and ovary-enriched in 02428-4x. The scale bar indicates the relative expression levels of miRNAs (log2). PMA, MA, SCP and BCP represent pre-meiotic interphase, meiosis and single microspore stage and bi-cellular pollen stage, respectively. MF, MM, FMF and EES represent megasporocyte formation stage, megasporocyte meiosis stage, functional megaspore formation stage and eight-nucleate embryo sac developing-stage, respectively. Up-regulation and Down-regulation represent the up-regulated and down-regulated miRNAs during embryo sac development compared to pollen development in autotetraploid rice.

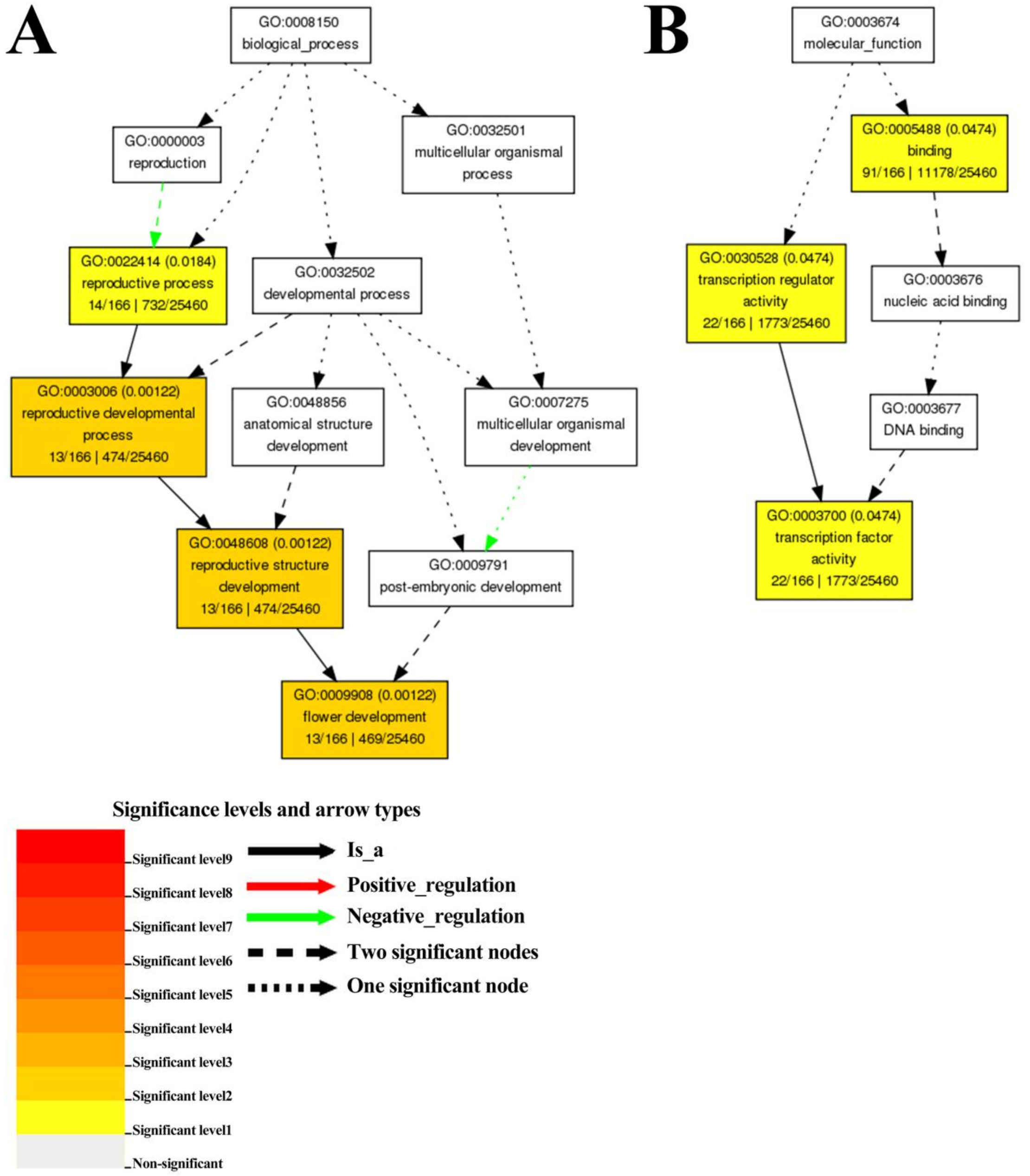

Fig. S9 GO (Gene Ontology) enrichment analysis of predicted targets of DEM-P (pollen-enriched miRNAs) in autotetraploid rice. (A) Biological process category; (B) Molecular function category. Arrows and shading are defined in the key.

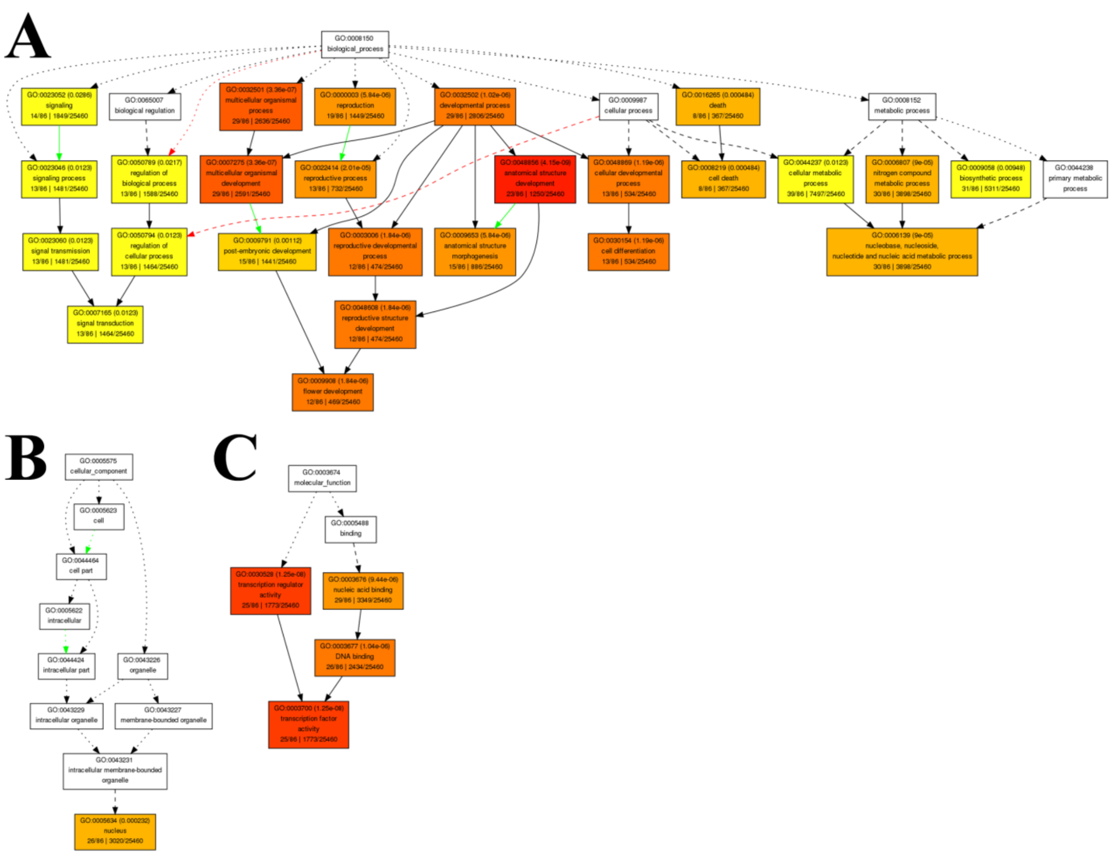

Fig. S10 GO enrichment analysis of predicted targets of DEM-ES (embryo sac-enriched miRNAs) in autotetraploid rice. (A) Biological process category; (B) Cellular component category; (C) Molecular function category. Arrows and shading are defined in the key in Fig. S9.

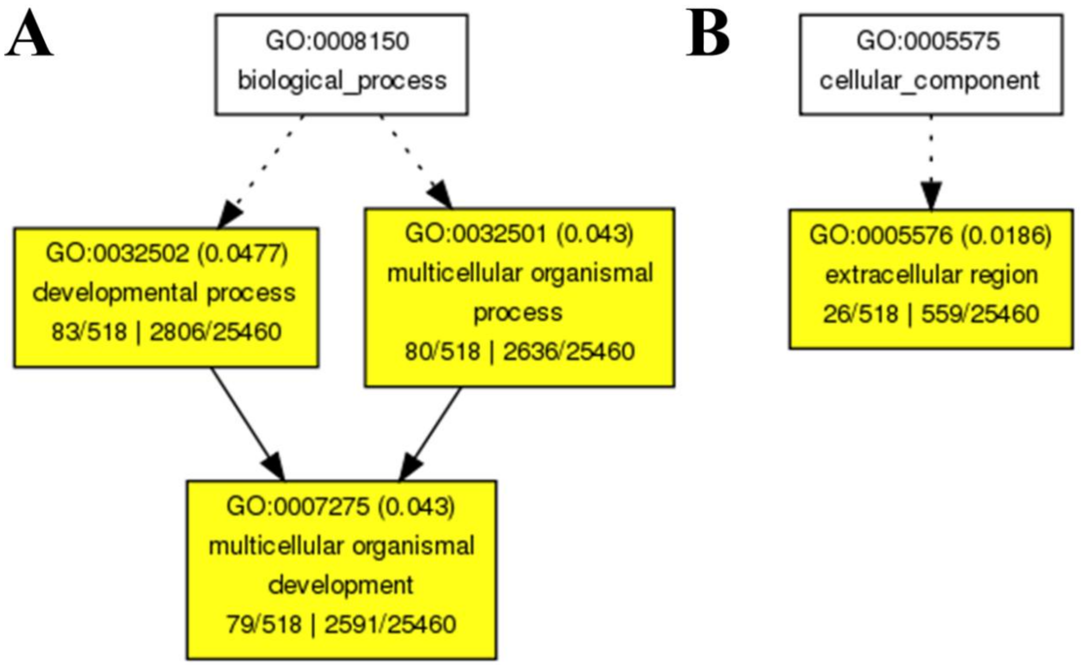

Fig. S11 GO enrichment analysis of predicted targets of differentially expressed miRNAs in MA (meiosis). (A) Biological process category; (B) Cellular component category. Arrows and shading are defined in the key in Fig. S9.

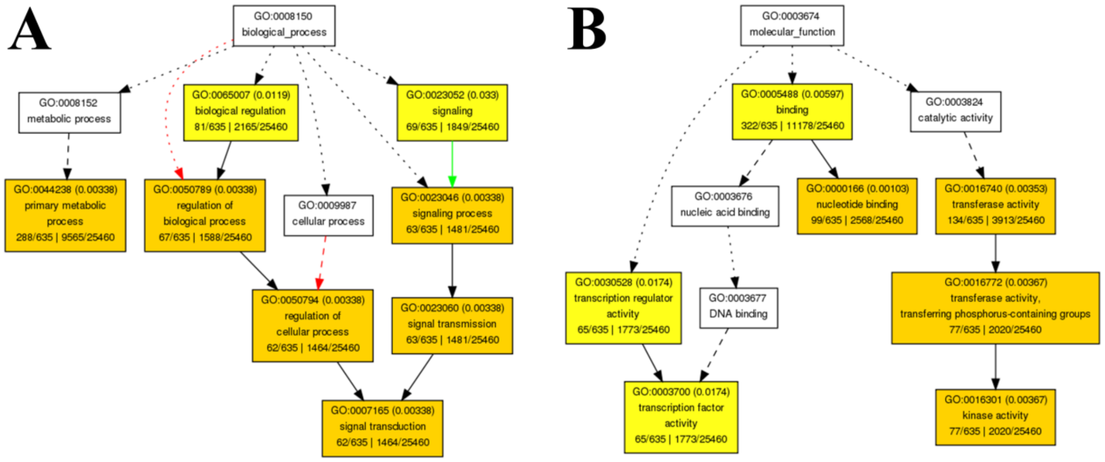

Fig. S12 GO enrichment analysis of predicted targets of differentially expressed miRNAs in MM (megasporocyte meiosis stage). (A) Biological process category; (B) Molecular function category. Arrows and shading are defined in the key in Fig. S9.

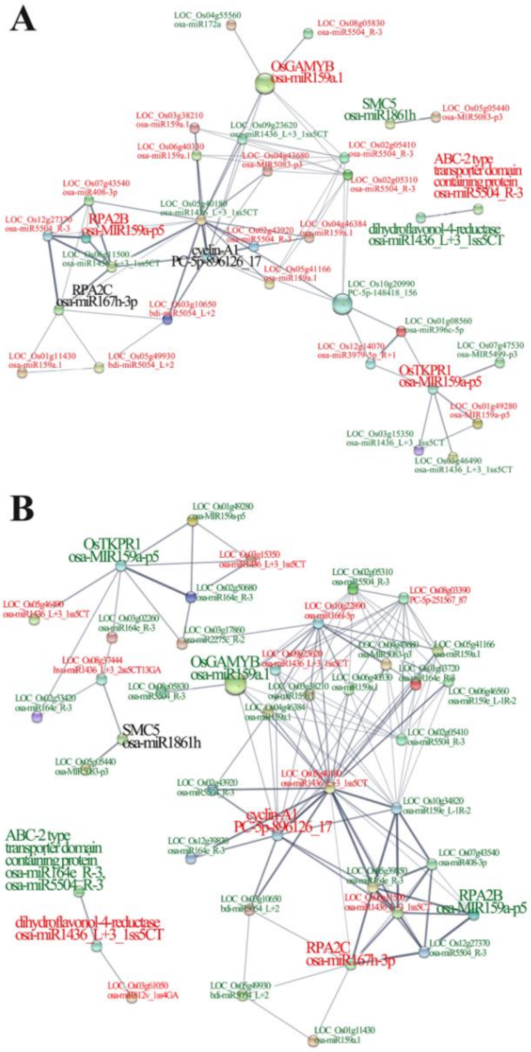

Fig. S13 Protein interactions between the targets of DEM (differentially expressed miRNAs) and meiosis-related genes. (A) Protein interaction in male meiosis. (B) Protein interaction in female meiosis. Red and green fonts represent the up- and down-regulated miRNAs in autotetraploid rice.

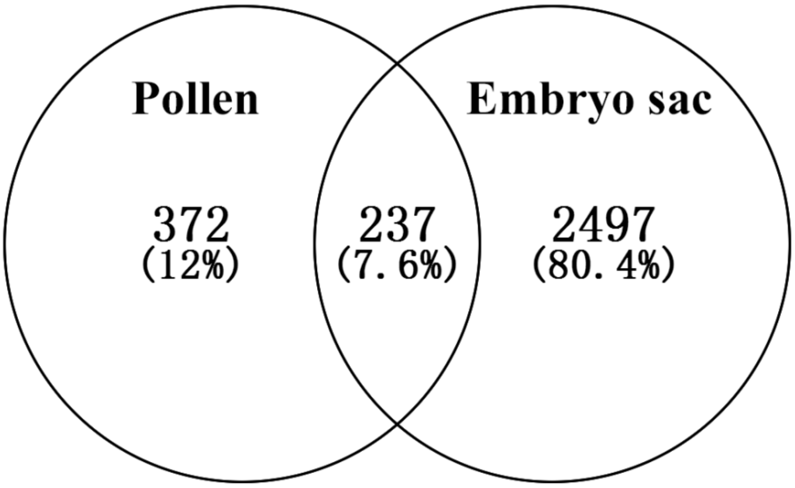

Fig. S14 Distribution of TEs-siRNAs (siRNAs associated with transposable elements) in pollen and embryo sac development.
